# Systems Approach to Pathogenic Mechanism of Type 2 Diabetes and Drug Discovery Design Based on Deep Learning and Drug Design Specifications

**DOI:** 10.3390/ijms22010166

**Published:** 2020-12-26

**Authors:** Shen Chang, Jian-You Chen, Yung-Jen Chuang, Bor-Sen Chen

**Affiliations:** 1 Laboratory of Automatic Control, Signal Processing and Systems Biology, Department of Electrical Engineering, National Tsing Hua University, Hsinchu 30013, Taiwan; s107061595@m107.nthu.edu.tw (S.C.); s107061588@m107.nthu.edu.tw (J.-Y.C.); 2 Institute of Bioinformatics and Structural Biology, National Tsing Hua University, Hsinchu 30013, Taiwan; yjchuang@life.nthu.edu.tw

**Keywords:** type 2 diabetes (T2D), pathogenic mechanism, deep neural network (DNN)-based DTI model, pathogenic biomarkers, drug design specification, multiple-molecule targeting drug

## Abstract

In this study, we proposed a systems biology approach to investigate the pathogenic mechanism for identifying significant biomarkers as drug targets and a systematic drug discovery strategy to design a potential multiple-molecule targeting drug for type 2 diabetes (T2D) treatment. We first integrated databases to construct the genome-wide genetic and epigenetic networks (GWGENs), which consist of protein–protein interaction networks (PPINs) and gene regulatory networks (GRNs) for T2D and non-T2D (health), respectively. Second, the relevant “real GWGENs” are identified by system identification and system order detection methods performed on the T2D and non-T2D RNA-seq data. To simplify network analysis, principal network projection (PNP) was thereby exploited to extract core GWGENs from real GWGENs. Then, with the help of KEGG pathway annotation, core signaling pathways were constructed to identify significant biomarkers. Furthermore, in order to discover potential drugs for the selected pathogenic biomarkers (i.e., drug targets) from the core signaling pathways, not only did we train a deep neural network (DNN)-based drug–target interaction (DTI) model to predict candidate drug’s binding with the identified biomarkers but also considered a set of design specifications, including drug regulation ability, toxicity, sensitivity, and side effects to sieve out promising drugs suitable for T2D.

## 1. Introduction

In recent years, chronic diseases are major causes of morbidity and mortality worldwide. As patients’ long-term conditions could deteriorate gradually with age, chronic diseases require continuous monitoring and treatment to maintain quality of life. Diabetes is one of the prominent chronic diseases caused by either dysfunctional insulin production or failed deployment of insulin. Among them, type 2 diabetes (T2D) accounts for 90% to 95% cases in all diabetes and is estimated to impact about 435 million patients around the world by 2030 [1]. While T2D is considered to be most common in adults, the diagnosis of pediatric T2D increases steadily [2]. Common symptoms for T2D include frequent urination, thirst, constant hunger, etc. Most importantly, as a risk factor for heart, blood vessels, eyes, kidneys, and nervous system diseases, T2D might inevitably increase the risk of death and the medical burden on society.

T2D has been typically seen as insulin-independent, which implies an ineffective utilization of insulin due to insulin resistance [3]. However, it has been shown that the pancreatic β-cell destruction due to inflammation and immune response might also give rise to T2D aggravation [4,5]. Nowadays, albeit much effort has been dedicated to elucidate the T2D pathogenic mechanism, few studies discussed how pancreatic destruction occurs in T2D, let alone its correlation with inflammatory response on β-cells. In fact, the systematic pathogenic mechanism of T2D still remains unclear. Therefore, we proposed a systems biology approach to investigate key pathogenic factors in view of genetic and epigenetic networks through system identification and system order detection methods by genome-wide RNA-seq data of T2D.

In the past decade, many methodologies have been proposed to identify the complex relations between the gene–gene, gene–protein, and protein–protein interactions. Although traditional biological experiments have been used to identify the protein–protein interaction network in the late 1990s [6], some drawbacks were also incurred. First, it is expensive and time-consuming to execute a large number of experiments for developing new therapies. Second, the biological experiments have practical limitations on taking the whole genome into consideration [7]. As a result, some potential pathways for diseases may not be well detected and studied. For instance, while the genome-wide association studies (GWAS) have investigated the single nucleotide polymorphisms (SNPs) of the human genome and found many disease-related variations, such findings alone can not explain the complex pathogenesis [8,9]. To overcome these challenges, we employed a systems biology approach to macroscopically analyze the systematic relationship among the proteins, genes, and microenvironment in the T2D pathogenic mechanism.

The systems biology method has been widely used to investigate the pathogenesis of disease such as cancer [10] and the progression of virus infection [11]. Likewise, in the proposed issues of T2D management, the systems biology method was deployed to trim off false positives from the candidate genome-wide genetic and epigenetic networks (GWGENs) as well as identify the disease-based GWGENs. Then, with the help of the principal network projection (PNP) approach, the core GWGENs were sifted out and further projected to KEGG pathways for subsequent analysis. Right after, by comparing the discrepancy between the non-T2D and T2D core signaling pathways, the pathogenic mechanism can be revealed. According to the analyzed pathogenic mechanism in core signaling pathways, the fat accumulation-dependent signaling pathways and the high glucose-induced signaling pathways lead pancreatic β-cells to excessive burden, bringing about cell inflammation and apoptosis during the development of T2D. Such phenomenon reduces the production of insulin secretion and disrupts the balance between glucose and insulin, giving rise to T2D. Hence, we proposed IKK, STAT3, PPARƔ, ETS1, and FAS as the significant pathogenic biomarkers contributing to the fat accumulation-dependent as well as the inflammatory-dependent cell apoptosis for systematic drug discovery design.

The process of drug development is an arduous task because of the high cost and time-consuming trials. It is estimated that it takes about 12–15 years and more than one billion US dollars before marketing a new drug [12]. Pharmaceutical companies need to spend a large amount of time and effort on executing experiments to understand the properties and the interactions of the drug to its targets. In addition, numerous animal and clinical trials ought to be carried out so as to ensure its safety and effectiveness [13]. These complicated procedures vastly increase the risk of failure in drug development, and most of them originate from the poor clinical outcomes [14]. On this ground, we developed systematic strategies based on drug–target interaction prediction and drug design specifications, which include drug regulation ability, toxicity, sensitivity, and side effect, to confront the problems from the perspective of system engineering. As the result, we chose and combined Sulforaphane and Biotin as the multiple-molecule targeting drug, which may potentially regulate IKK, STAT3, PPARƔ, ETS1, and FAS for T2D management. Taken together, we expect that the systematic drug discovery and design procedures presented in this study can provide an efficient way to find the multiple-molecule targeting drug candidates for T2D treatment before clinical trials.

## 2. Results

### 2.1. Overview of Systems Biology Method and Systematic Drug Discovery Design in T2D

**Figure 1:**
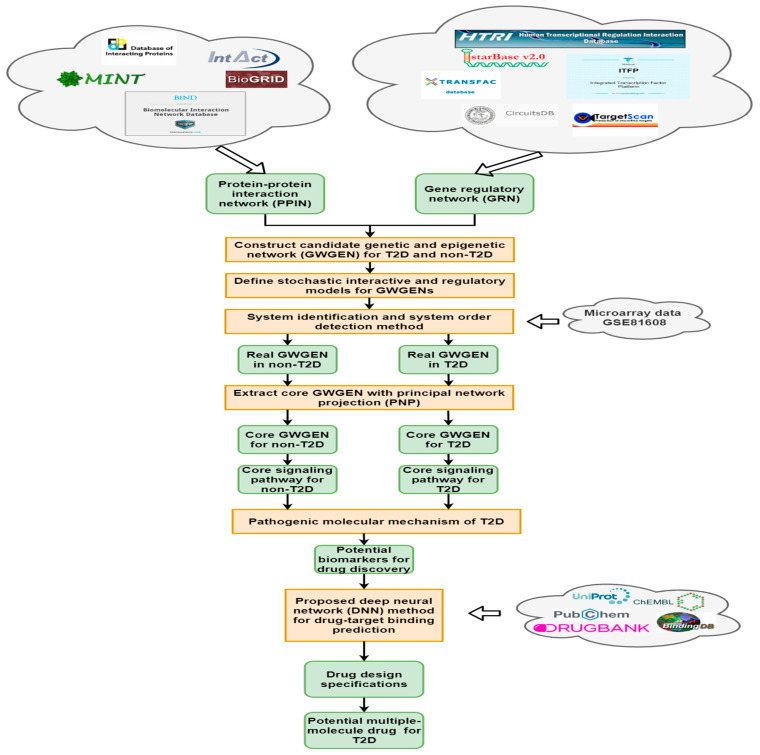
Flowchart of systems biology method and the outline of systematic drug discovery design. The candidate genome-wide genetic and epigenetic networks (GWGEN) consist of gene regulation network (GRN) and protein–protein interaction network (PPIN), where candidate GRN was constructed through integrating gene regulation databases and candidate PPIN was constructed via protein–protein interaction databases. The candidate GWGENs were identified to obtain real GWGENs by RNA-seq data from GSE81608 through system identification and system order detection. Then, core GWGENs were extracted from real GWGENs by the principal network projection (PNP) method. Potential drugs were discovered according to the significant biomarkers determined by investigating the T2D pathogenesis constructed through comparing core signaling pathways of non-type 2 diabetes (T2D) and T2D.

**Figure 2:**
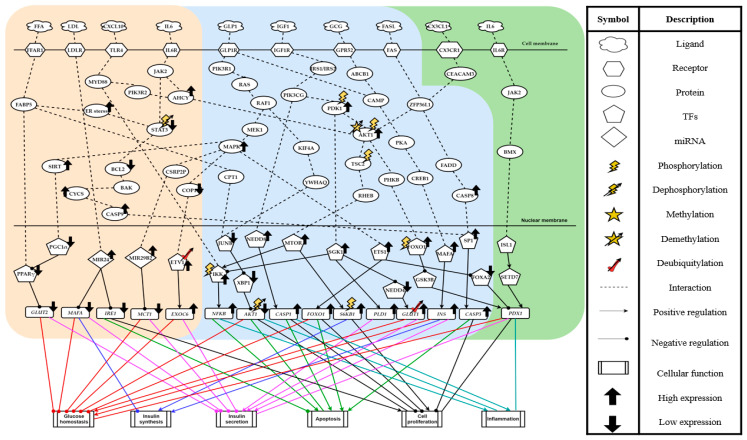
The T2D pathogenic mechanism investigation by comparing the T2D and non-T2D core signaling pathways. The genes and proteins in the core signaling pathways were chosen from the T2D and non-T2D core GWGENs. The gene regulations and protein interactions were constructed based on the edges in core GWGENs. The blocks of light orange, light blue, and light green background color separately indicate the T2D differential signaling pathways, the common signaling pathways of both T2D and non-T2D, and the non-T2D differential signaling pathways, respectively. The cellular functions caused by target genes are clustered with solid lines in different colors and referred to Uniprot. The bold arrowhead marks in black denote the relatively low and high expression in pathogenic signaling pathways in contrast to non-T2D.2.2. The Pathogenic Microenvironment in T2D.

**Figure A1:**
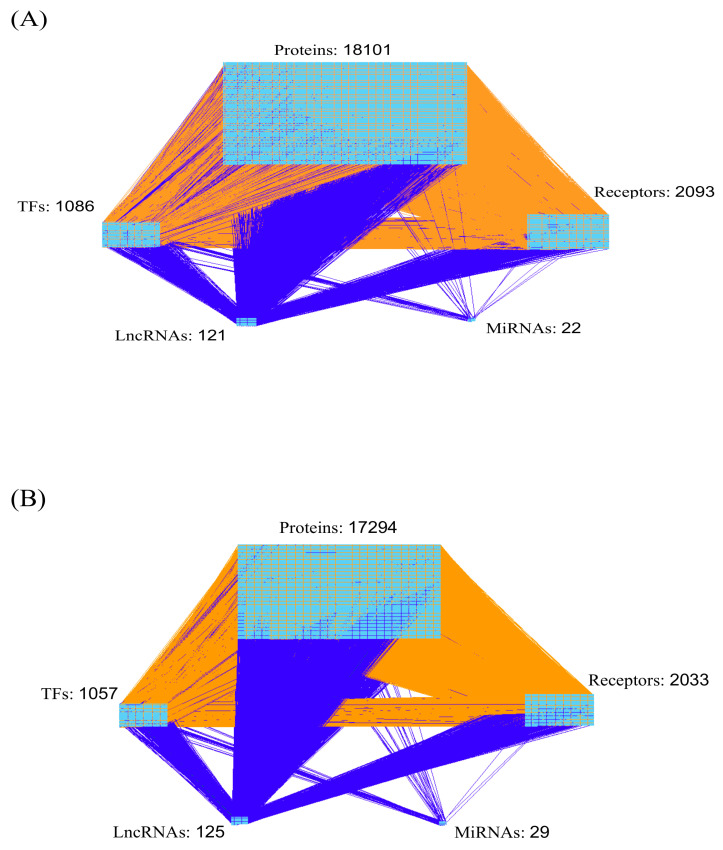
(**A**) The real GWGEN network of non-T2D; (**B**) The real GWGEN network of T2D. The real GWGENs were constructed by pruning the false positives from candidate GWGENs though system identification and systems order detection methods. The numbers in the figure signify the node numbers of proteins, TFs, Receptors, LncRNAs, and MiRNAs. The orange lines indicate the protein–protein interactions and the blue lines denote the gene regulations.

**Figure A2:**
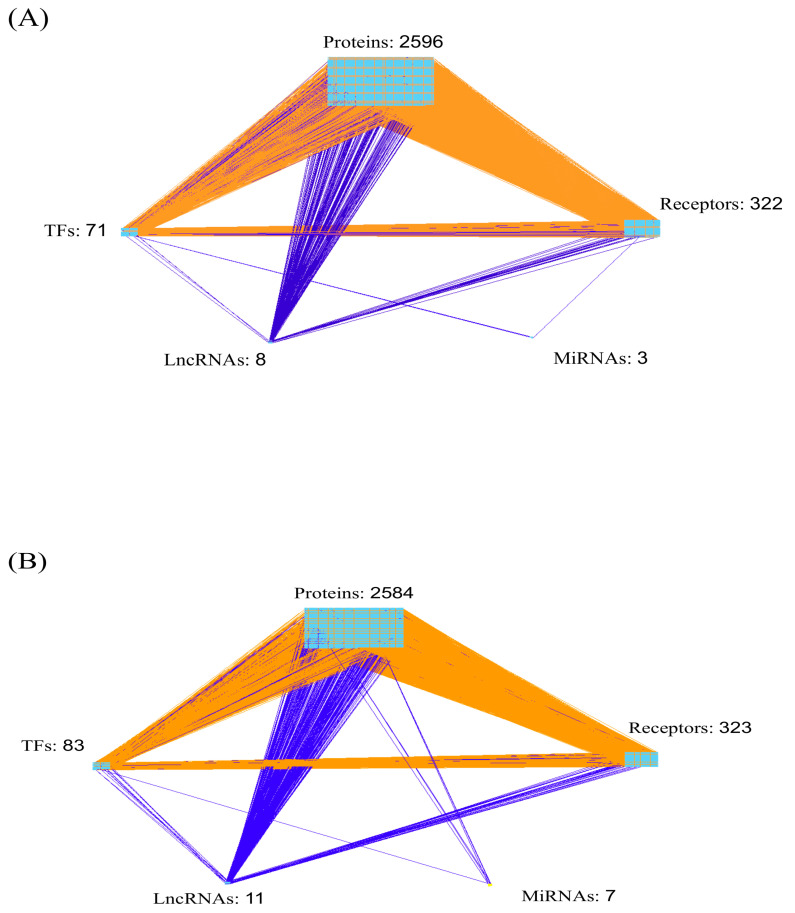
(**A**) The core GWGEN network of non-T2D; (**B**) The core GWGEN network of T2D. The core GWGENs were extracted by PNP method the real GWGEN to simplify the pathogenic analysis of T2D. The numbers in the figure signify the node numbers of proteins, TFs, Receptors, LncRNAs and MiRNAs. The orange lines indicate the protein–protein interactions and the blue lines denote the gene regulations.

In this work, we proposed a combination of systems biology method and systematic drug discovery design (as shown in Figure 1) to gain deeper insight into the T2D pathogenesis and to identify potential drugs for T2D treatment based on the selected significant biomarkers (drug targets). By and large, the process can be subdivided into a few steps: (1) candidate GWGENs construction from big data mining; (2) the system identification method by RNA-seq data and system order detection method to construct real GWGENs shown in Figure A1 by pruning the false positives from candidate GWGEN; (3) the principal network projection method (PNP) for extracting core GWGENs shown in Figure A2 from the real GWGENs to simplify the network analysis; (4) the pathogenic mechanism of T2D and the significant biomarkers investigating by comparing the core signaling pathways between non-T2D and T2D in Figure 2; (5) a pretrained drug–target interaction (DTI) model to predict candidate drugs for the targets (biomarkers); (6) drug design specifications to further sieve out promising drugs for the proposed drug combination (multiple-molecule targeting drug).

Note that, to reinforce the reliability of constructed T2D pathogenic mechanism, the collected RNA-seq data on the pancreatic β-cell was selected with age greater than or equal to 50 years due to high incidence, and they were classified into non-T2D and T2D, as shown in Table 1.

Based on the information from the accessible bioinformatics databases, the candidate GWGENs were constructed and identified by system identification and the system order detection method to prune off the trivial interactions and regulations. Although the extracted GWGENs (real GWGENs plotted by Cytoscape software in Figure A1) in a smaller scale could be apparently observed as shown in Table A1, the network complexity it owns still blocked the further analysis. To deal with this problem, the PNP method was applied to distill the real GWGENs into the core GWGENs, which effectively reduced the network size and simplified the subsequent pathogenic markers and pathway analysis of T2D. Notably, among the core GWGENs as shown in Figure A2, the top 3000 major nodes from 85% of the real GWGENs after projection were included.

**Figure A3:**
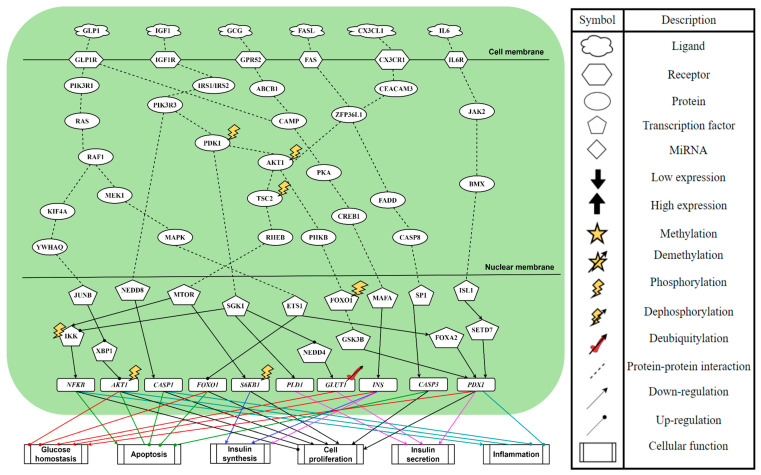
The core signaling pathways of non-T2D based on our result for investigating the non-T2D genetic and epigenetic molecular mechanism. The genes and proteins in the core signaling pathways were chosen from the non-T2D core GWGENs. The gene regulations and protein interactions were constructed on the basis of the edges in non-T2D core GWGENs. The cellular functions caused by target genes are clustered with solid lines in different colors.

**Figure A4:**
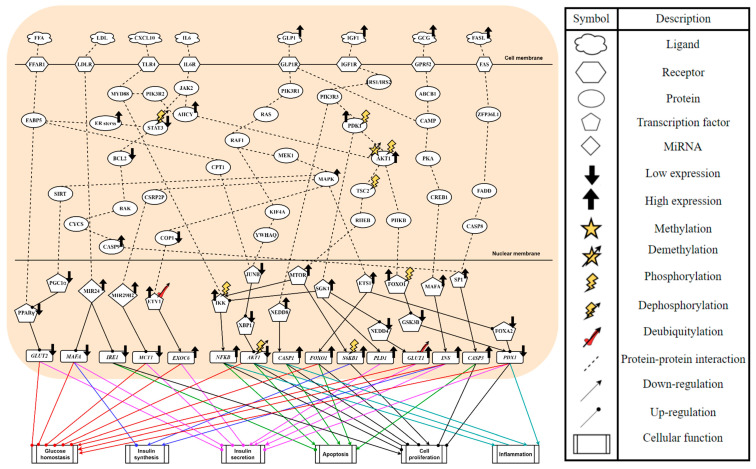
The core signaling pathways of T2D based on our result for investigating the T2D genetic and epigenetic molecular mechanism. The gene regulations and protein interactions were constructed on the basis of the edges in T2D core GWGENs. The cellular functions caused by target genes are clustered with solid lines in different colors. The cellular functions caused by target genes are clustered with solid lines in different colors. The bold arrowhead marks in black denote the relatively low and high expression in T2D pathogenic signaling pathways in contrast to non-T2D.

Thereafter, the core GWGENs for T2D and non-T2D were projected to KEGG pathways by DAVID software to derive the core signaling pathways in Table 2 and Table 3, respectively. According to the enrichment analysis of core T2D signaling pathways shown in Table 2, there are 22 genes related to insulin resistance and 11 genes related to type II diabetes mellitus. In addition, 14 genes are associated with lipid metabolism. Such findings indicate that pathogenic factors of diabetes are related to not only high glucose but also fat accumulation. As a result, with the help of KEGG pathway annotation, core signaling pathways for T2D and non-T2D were constructed and individually illustrated in Figure A3 and Figure A4.

According to the T2D pathogenic signaling pathways (Figure 2), the lipid and glucose metabolism pathways are found to play crucial roles in impairing the pancreatic functions, leading to the occurrence of T2D. Lipid accumulation in the pancreas owing to long-term excessive caloric intakes inevitably causes the burden on the pancreas β-cell, which thereby impacts the insulin production. In our body, lipids are degraded into either the triglycerides or the free fatty acids (FFAs). Some of them might also transform into low-density lipoproteins (LDLs). It is known that LDLs and FFAs can act to interfere with insulin biosynthesis, insulin secretion, and cell proliferation [15]. On the other hand, serving as a peptide hormone to consolidate the concentration of glucose level in blood and to stimulate the decomposition of fat, glucagon (GCG) would be suppressed to a lower concentration due to the high glucose intake. Therefore, the ability of lipid decomposition is declined. In addition, higher glucose is often accompanied by the enrichment of glucagon-like peptide-1 (GLP1) and insulin-like growth factor 1 (IGF1). Although the provoked downstream pathways may expand the cell mass and enhance the capacity of insulin secretion to balance the blood sugar level, extreme glucose intake often disturbs homeostasis. As a result, the pancreatic β-cells cannot withstand the impact of high glucose and lipids, and they eventually cause dysfunction.

Furthermore, the effect of immune and inflammatory responses should not be neglected. When the pancreatic β-cells suffer damage from endoplasmic reticulum stress (ER stress), the immune response would be activated along with the secretion of cytokine factors, such as IL6 and FAS or chemokine CXCL10.

### 2.2. Pancreatic β-Cell Proliferation and Apoptosis in the T2D Inflammatory Microenvironment

We conducted a literature survey to outline the biological functions implied in Figure 2. Under high glucose conditions, GLP1 and IGF1 ligands were induced to a higher level than normal. Catalyzed by GLP1, GLP1R delivered the transduction signal via PIK3R1, RAS, RAF1, and MEK1 to activate the MAPK pathway. The activated MAPK due to the overexpression of GLP1 obliquely elevated the level of transcription factor (TF) ETS1, which subsequently upregulated the target gene *FOXO1* but downregulated TF FOXA2. Emerging studies revealed that the upregulation of *FOXO1* contributes to the apoptosis of the pancreatic β-cell, concurrently alleviating cell proliferation [16]. In addition, serving as an important TF in pathogenic pathways, FOXO1 could suppress TF GSK3B to elevate *PDX1* expression, where GSK3B is a negative regulator, and its downregulation maintains PDX1 protein stability to delay its phosphorylated degradation [17]. As a critical regulator in pancreatic β-cell development, PDX1 is responsible for cell proliferation and insulin secretion [18]. However, it has been reported that a decreased FOXA2 could reduce its binding to the *PDX1* promoter [19], holding an antagonism. If without sufficient PDX1, pancreatic β-cells cannot repair the damage from cell apoptosis and peroxide [20]. It has been validated that the AKT1 activates the downstream protein MTOR through TSC2 and RHEB and simultaneously upregulates TF FOXO1, which is phosphorylated by kinase PHKB [21,22]. In T2D, MTOR phosphorylated S6KB1, and it is well documented that this upregulation expands the cell size and number of pancreas for producing more insulin and maintaining the pancreatic function to decompose the glucose [23]. Among signaling transductions associated with pancreatic β-cell survival, upon receiving the signal from ligand low-density lipoprotein (LDL), receptor LDLR stimulated MIR24 to restrain the transcription of target gene *IRE1*. Notably, the inhibition of *IRE1* protects the pancreatic β-cell from ER stress-induced apoptosis while accelerating the impairment of insulin secretion [24].

Furthermore, the influence of AKT1-dependent immune response, FFA-induced, and MAPK-relevant pathways occupied a key position on cell survival. In the AKT1 pathway, PDK1 suppressed TF IKK phosphorylation degradation through SGK1. SGK1 plays a role in anti-inflammation, since it impedes the apoptotic promoter *NF-κB* from translocation to mitigate its ability for inflammatory cytokines transcription [25]. In contrast, the MTOR pathway and CXCL10-mediated MYD88 signaling both enhanced the nuclear translocation of *NF-κB* through IKK. It can initiate immune response, contributing to the cell inflammation and apoptosis [26,27]. *NF-κB* is a double-edge sword in immune modulation. In general, *NF-κB*-dependent transcription not only accelerates the anti-apoptosis mechanism in favor of cell survival but also augments the inflammatory response, leading to cell death. Nevertheless, in T2D, the absence of anti-apoptosis pathways pertaining to *NF-κB* was found.

On top of that, FFA mediated the reduction of AKT1 phosphorylation by JUNB and XBP1, hence weakening the transcriptional ability of *AKT1*.

Concerning cell death, the members of the CASP family count for a great deal in inducing apoptosis when stimulated by exogenous and endogenous environmental factors. The IGF1 signal indirectly induced the NEDD8 activation and further upregulated the inflammatory mediator CASP1, resulting in an aggravated inflammation response [28]. Another CASP member, CASP8, could be triggered by the FASL-stimulated apoptotic pathway as well, causing the inception of cell inflammatory response and apoptosis. On the other hand, as a typical hallmark of apoptosis in the CASP family, CASP3 could be indirectly activated by IL6. Although IL6 might upregulate AKT1 activity to raise the cell proliferation through JAK2-induced demethylation, IL6 inhibited the key controller of anti-apoptosis BCL2 through STAT3 downregulation. It is worth noting that from the non-T2D signaling pathway, IL6 was characterized as an anti-inflammatory cytokine and indirectly interacted with ISL1 and SETD7 to activate *PDX1*, intensifying cell proliferation. Moreover, the FFA-dependent ER stress could also interrupt the STAT3-dependent signaling, which causes the blockade of cellular defensive machinery from BCL2. The inhibited BCL2 activated the caspase cleavage TF SP1 and obliquely its target gene *CASP3*, resulting in apoptosis, which has been suggested through opening the channel on mitochondria membrane to secret CYCS [29].

### 2.3. Abnormality in Insulin Synthesis and Insulin Secretion

Insulin synthesis and secretion are significant and indispensable modulation functions in the pancreas. Without sufficient insulin, the pancreas is not able to effectively decompose glucose to generate enough energy for cell tissues. In T2D, there exist confrontations between the glucose-induced promotion of insulin and the apoptosis-triggered reduction of insulin secretion. From the pathogenic signaling pathways shown in Figure 2, the high expression of phosphorylated PDK1 interacted with TF SGK1 to prompt the upregulation of *PLD1*, which has previously been described to facilitate insulin secretion [30]. Likewise, the increment of insulin production could also be triggered by GCG-stimulated TF MAFA activation pathway through signaling cascades GPR52, ABCB1, CAMP, PKA, and CREB1. This finding is in line with the observation of a study that GCG level rises in response to lipid metabolism when lipids accumulate in the pancreas [31]. Furthermore, the upregulated SGK1 inhibited NEDD4 to accelerate the *GLUT1* deubiquitylation, promoting the insulin secretion. On the other hand, as the ubiquitin ligase, COP1, its inhibition induced by the GLP1-stimulated MAPK pathway could attenuate the degradation of negative modulator ETV1 and impel its target gene *EXOC6* to overexpress, therefore weakening the ensuing insulin secretion stimulation.

Meanwhile, in contrast to the upregulation of *GLUT1*, the target gene *GLUT2* was repressed by PPARγ through both the signaling cascades: one via FFA-dependent FFAR1 and FABP5 signal transduction; the other via the decrement of TF PGC1α transcriptional ability through GLP1-catalyzed deacetylated enzyme SIRT upregulation. Consequently, the loss of *GLUT2* gave rise to a drop on insulin secretion. Furthermore, miRNAs also play a key role in the pancreas to regulate insulin synthesis and secretion. An abnormal expression of miRNAs often arouses repercussion. Despite holding the potency to prevent pancreatic β-cells from apoptosis through *IRE1* inhibition [24], MIR24 was inevitably induced by LDL to dampen insulin synthesis through triggering *MAFA* downregulation. Aside from that, acting as a downstream of MAPK signaling cascades, when activated, MIR29B2 kept its target *MCT1* (*SLC16A1*, a plasma membrane monocarboxylate transporter to manage the exocrine function of insulin) from expression, thereby resulting in the interruption of insulin secretion [32].

### 2.4. Potential Multiple-Molecule Targeting Drug for T2D Utilizing Systematic Drug Discovery Approach

According to the investigation of the pathogenic mechanism, the primary progression of T2D stemmed from excessive inflammation and cell apoptosis owing to fat accumulation in the pancreas. Moreover, an over intake of glucose pressures the pancreas to overwork, therefore leading to dysfunction. In line with this notion, significant biomarkers related to fat accumulation, cell inflammation, and apoptosis were selected. Then, we used these biomarkers to search for favorable compounds that can serve as potential therapy of T2D. Consequently, we took IKK, STAT3, FAS, ETS1, and PPARγ as biomarkers and sought to reverse their expression levels. Amongst them, IKK, STAT3, and FAS are pertinent to pancreas inflammation and death; ETS1 is responsible for pancreas proliferation; PPARγ can regulate the glucose flux into the pancreas through the channel protein GLUT2 and therefore stimulate insulin secretion.

**Figure 3:**
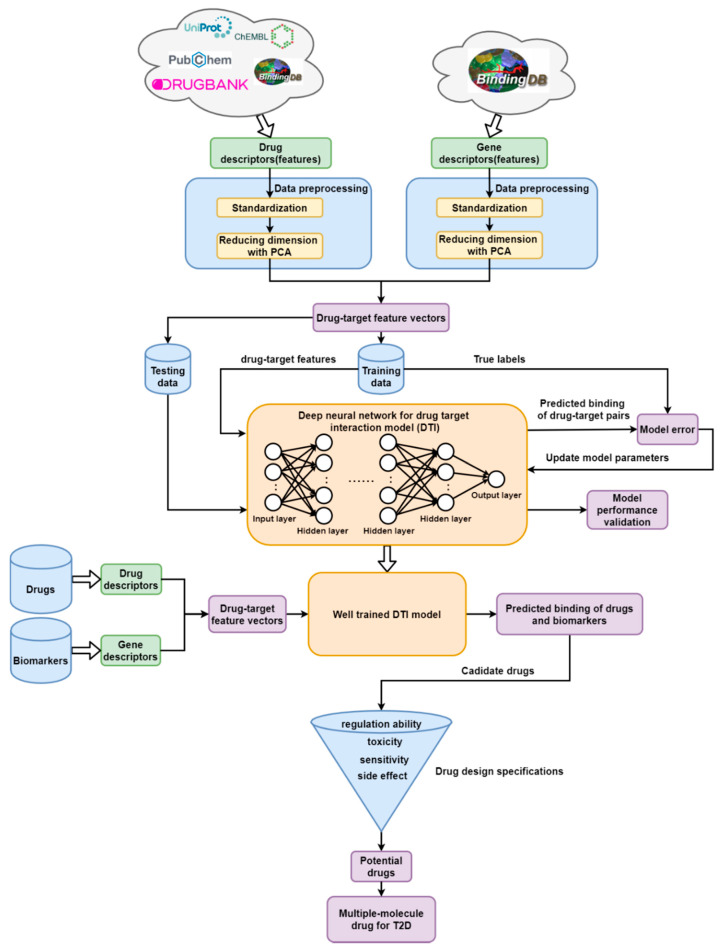
The flowchart of systematic drug discovery and design procedure. The drug–target binding datasets were obtained from BindingDB, which integrated substantial information of drugs and targets from several databases. Then, the drug and target features were sequentially preprocessed through descriptor transformation, standardization, and PCA dimension reduction. Afterwards, the processed data were split into training and testing data for deep neural network (DNN)-based drug–target interaction (DTI) model training and performance evaluation, respectively. During the training process, the model parameters were updated through the error between the true binding label and predicted binding label of each drug–target pair. The well-trained DNN-based DTI model was used to predict the binding probability between drugs and the identified biomarkers to sift out candidate drugs. Finally, with the consideration of drug design specifications including regulation ability, toxicity, sensitivity, and side effect, potential drugs were selected and integrated for novel medication therapy curing T2D.

After defining these potential biomarkers as drug targets, we select candidate drugs by drug repositioning, with consideration of their chemical properties. On one hand, a deep neural network (DNN)-based DTI model was pretrained to predict drug–target binding likely to exist; on the other, drug design specifications, i.e., regulation ability, toxicity, sensitivity, and side effect were further exploited to sieve out potential drugs for designing a multiple-molecule targeting drug for T2D treatment before clinical trials. The flowchart of systematic drug discovery and design procedure is described in Figure 3.

**Figure 4:**
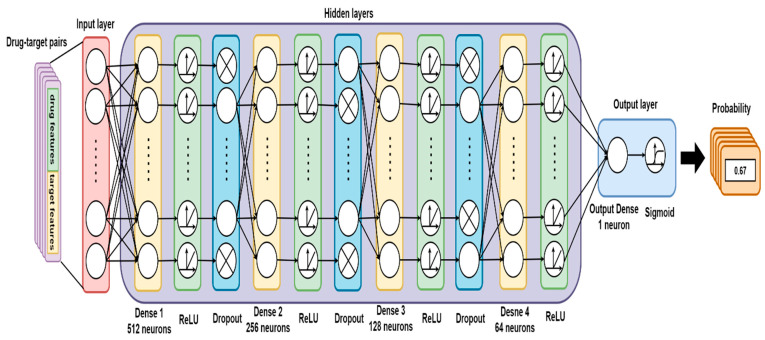
The schematic diagram of the DNN-based DTI model.

**Figure 5:**
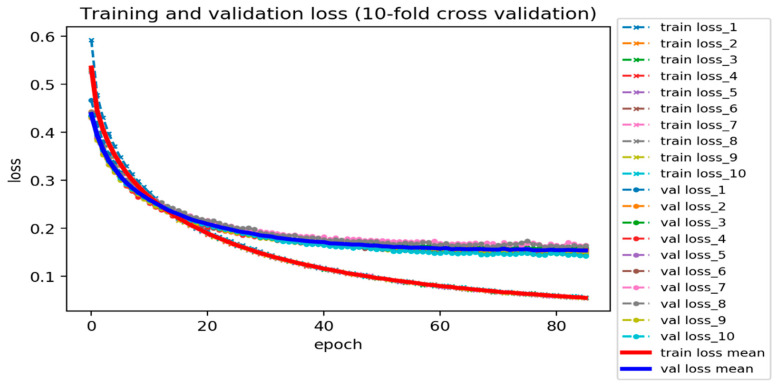
Training and validation loss of DNN-based DTI model (10-fold cross-validation).

**Figure 6:**
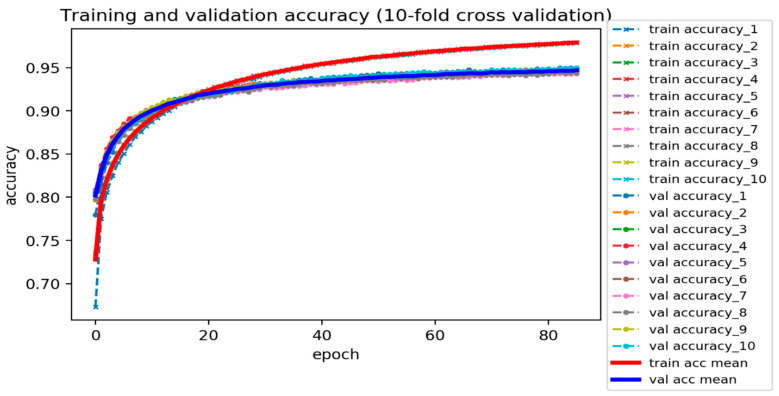
Training and validation accuracy of DNN-based DTI model (10-fold cross validation).

From our DNN-based DTI model (Figure 4), we set four hidden layers, and each of them is connected with a ReLU activation function layer behind. The ReLU activation function could avoid vanishing gradient problems and converge much faster than the other activation functions adopted to deal with classification issues [33]. Meanwhile, to hinder the model from overfitting during the training process, the dropout layer is incorporated after each hidden layer. The dimension of the input layer is 618, corresponding to the features size of the drug–target pair, and 512, 256, 128, and 64 neurons are embedded respectively in the four hidden layers. Prior to the output layer, a sigmoid activation function is applied to limit the value within the range between 0 and 1 (probability). Note that a sigmoid function is commonly used in binary classification problems. The outcome of DTI indicates the likelihood of a binding, where a higher value corresponds to a more reliable interaction (binding) between the drug and target. The loss and accuracy during the training process are recorded in Figure 5 and Figure 6, respectively. The well-trained DTI model was supervised through applying the 10-fold cross-validation to evaluate the model performance, as shown in Table 4. Eventually, we received an average accuracy of 94.89 (%) with the standard deviation of 0.156 (%), and the model with best testing performance was picked as our DTI model.

**Figure 7:**
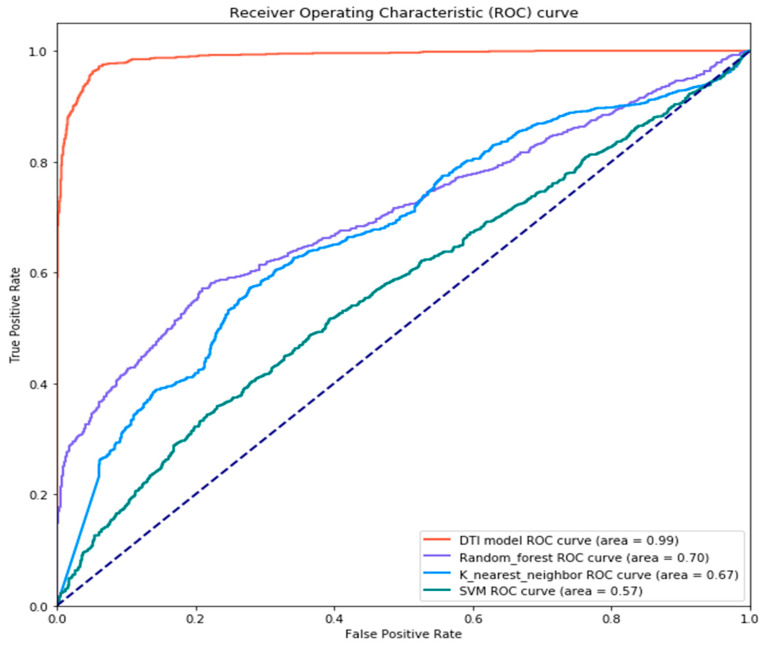
The receiver operating characteristic (ROC) curves and AUC (area under the curve) scores for DTI model based on DNN, random forest, K-nearest neighbor (KNN) and Support Vector Machine (SVM). The dotted line on the diagonal indicates the virtual model without predicted value due to random prediction and is the boundary for judging whether the model performs well. On the upper left of the dotted line, the model is better than the randomly predicted model; contrarily, on the right lower of the dotted line, the model is worse than the randomly predicted model. The “area” in the parenthesis of each label denotes the AUC score.

Furthermore, we also compared the DNN-based DTI model with other DTI models based on machine learning classification approaches, such as random forest, K-nearest neighbor (KNN), and Support Vector Machine (SVM) by the receiver operating characteristic (ROC) curve measure. The visualization of ROC curve comparison is denoted in Figure 7. From the figure, the performance of our proposed DTI model is apparently better than the others, which indicates that the deep learning algorithm greatly adapts to the calculation of the overwhelming and complicated drug–target interaction data in contrast to other traditional machine learning methods.

Through the prediction of the pretrained DNN-based DTI model, candidate drugs were sieved out owing to possessing high probability to bind (dock) to the selected biomarkers. However, the balance between the drug potency and adverse effect should also be concerned, since potent drugs are usually accompanied with a high risk of damage. Accordingly, with the consideration of the drug design specifications such as regulation capacity, toxicity, sensitivity, and side effect, we could further assure the stability and safety of drugs in clinical trials. For the purpose of measuring the regulation capacity of candidate drugs, the available data with well-documented regulation ability information was downloaded from L1000 level 5 dataset, which contains 978 genes treated with 19,811 small molecular compounds in 78 different cell lines [34]. By referring to LINCS L1000, we can examine whether a specific gene was upregulated (positive values) or downregulated (negative values) after being treated with an existing compound. On the other hand, the drug with lower toxicity often possesses a smaller side effect with reference to the median lethal dose (LD50) value in DrugBank. Being the numeric index of lethality, LD50 plays a pivotal role in drug safety evaluation. Further, administering a drug with higher drug sensitivity (a lower value of half maximal effective concentration (EC50)) could also cut down the dosage of the drug and further mitigate the ensuing side effect [35]. Within, the drug sensitivity data were collected from the PRISM dataset, which includes 4518 drugs being experimented across 578 human cell lines based on the EC50. EC50 is used to measure the potency of a drug, where a drug with smaller EC50 implies that it could exert the maximum effect with a lower dose [36]. On top of that, we defined the side effect of each drug as its additional binding to other targets rather than the desired biomarkers. The fewer unwanted targets the drug binds, the smaller it affects other pathways. The side effects for the candidate drugs are denoted in Table 5, and further information of the proposed candidate drugs for the identified biomarkers were presented in Table 6. Leveraging these pharmacological properties from databases, appropriate drugs were plausibly selected from Table 5 and Table 6 to meet the drug design specifications. Ultimately, we suggested a combination of Sulforaphane and Biotin as our potential multiple-molecule targeting drug for T2D.

Sulforaphane is a natural edible substance isothiocyanate produced by the enzymatic action of the myrosinase on glucopharanin, which is a 4-methylsulfinylbutyl glucosinolate contained in cruciferous vegetables of the genus Brassica such as broccoli, brussel sprouts, and cabbage. Several experiments have validated that Sulforaphane mitigates oxidative stress and protects cells from damage by invaded tumors and diseases [37]. Biotin, also called vitamin H, is a water-soluble B vitamin and involves a wide range of metabolic processes in body. It plays an important role in not only the protein synthesis but also the fat and carbohydrate metabolism. A previous experiment in rats documented that the insulin secretion dysfunction is related to the loss of biotin [38]. The chemical structures of the T2D multiple-molecule targeting drug and the corresponding drug design specifications with respect to suitable regulation ability, low toxicity, high sensitivity and low side effect are given in Table 7.

According to Table 5, Table 6 and Table 7, the combination therapy of Sulforaphane and Biotin has the potential to restore the abnormal regulation in T2D. The reversing of STAT3 may reduce the cell apoptosis caused by the endogenous damage substances, and the lower expression of FAS can decrease the cell apoptosis by the interference of an exogenous microenvironment. In addition, the reduction of IKK expression can suppress some phosphorylated degradations, hence mitigating the formation of inflammatory environments and the subsequent activation of cell apoptosis. Furthermore, the downregulation of ETS1 by the proposed multiple-molecule targeting drug can facilitate FOXA2 to form the connection with FOXO1, therefore enhancing the cell proliferation. The recovery expression of PPARγ can achieve the equilibrium between glucose intake and insulin secretion. Taken together, by administering the proposed multiple-molecule targeting drug, an upregulation of STAT3 and PPARγ accompanied by the downregulation of IKK, ETS1, and FAS can validly be attained, yielding encouraging results for the treatment of T2D patients.

## 3. Discussion

### 3.1. The Association between Macrophage Polarization and Inflammatory Response in T2D

When it comes to the T2D pathogenic mechanism, the pancreatic β-cell could not withstand the decomposition of excessive glucose in the body from long-term high glucose intakes, leading to pancreatic β-cell exhaustion and insulin resistance. Although the accumulation of glucose in the body is a crucial factor for T2D development, it is worth noting that the inflammatory-dependent apoptosis stemming from the fat accumulation in the pancreatic β-cell is also a pivotal issue. In T2D specific signaling pathways, FFA produced by the hydrolysis of oils and fats not only disrupted the glucose homeostasis but also increased the ER stress to indirectly trigger the follow-up inflammatory response, i.e., IKK-induced *NF-κB* pathway and apoptotic pathways related to the CASP family. Additionally, to form the inflammatory microenvironment, the immune response is initiated to activate the releasing of pro-inflammatory cytokines such as IL-1β and IL-6 when damage and infection occur.

Among all types of immune cells, macrophages exert a significant effect on pancreatic β-cells in T2D. Macrophages are mononuclear phagocytic cells and widely distributed in human organs. They play an indispensable role in physiological homeostasis, immune surveillance, and cell regeneration. There are mainly two types, M1- and M2-type macrophage, existing in the pancreas. M1-type macrophages are referred to as “pro-inflammatory macrophages” that can activate inflammatory response and recruit T-cells and natural killer cells to eliminate the harmful substance [39]. However, excessive inflammation stimulated by cytokine and chemokine often inevitably causes great harm to health. Conversely, M2-type macrophages named “anti-inflammatory macrophages” can primarily mediate the side effect arising from the inflammatory and immune response [40]. Under a high glucose and fat environment, glucotoxicity and lipotoxicity in response to the damage infringe the stability of pancreatic β-cell, so that the accumulation of intracellular stress including the oxidative and ER stress intensifies [41]. Moreover, the intracellular stress can modulate the polarization of macrophage from M2-type to M1-type, causing the imbalance in the ratio of M1-type/M2-type macrophages. Consequently, the number of M1-type macrophages residing in the pancreatic β-cell outweighs that of M2-type macrophages to induce pancreatic β-cell toward inflammatory-dependent apoptosis and pancreatic function impairment, which further strengthens the investigation of pancreatic β-cell destruction by apoptosis and inflammation in the T2D pathogenic mechanism.

### 3.2. The Modulation of Ion Channels Involved in Insulin Resistance and Glucose Homeostasis

Nutrients and chemical substances are essential to sustain cell stability and survival. The ways for substances intakes from the extracellular space into cells consist of the diffusion across the plasma membrane and the transmission on it via channel proteins. As modulators of ion channels residing on the plasma membrane, the GLUT family, i.e., GLUT1 and GLUT2, stood out as crucial factors to maintain the equilibrium between the glucose uptake and insulin secretion in the T2D pathogenic mechanism. As the result of high glucose intakes, GLUT1 and GLUT2 increase the ratio of ATP/ADP, leading to inducing the electrical and transductive signal to inactivate the KATP^+^ ion channel protein, a modulator of K^+^ flux in cells [42]. Then, the inactivated KATP^+^ channel further activates the opening of the Ca^2+^ ion channel, elevating the concentration of Ca^2+^ to promote insulin secretion [43]. However, in the aforementioned pathogenic signaling pathways of T2D, FFA-dependent pathway inhibited *GLUT2* to reduce the sensitivity of insulin secretion via pancreatic β-cell in response to glucose accumulation. This phenomenon has also been reported in diseases related to glycogen metabolism and metabolic disorders [44]. It is also commonly found in T2D and should be taken into consideration when performing its medical treatment.

### 3.3. Potential Multiple-Molecule Targeting Drug for to the Identified Biomarkers of T2D

In recent years, pharmacology companies have devoted to the discovery and design of drugs for T2D treatment, e.g., Metformin, Sulfonylureas, Meglitinides, Thiazolidinediones, DPP-4 inhibitors, GLP-1 receptor agonists, etc. Metformin is the first-line medication for the treatment of T2D, working for lowering the production of glucose in liver and improving insulin sensitivity. In spite of holding promising efficacy, it can give rise to acute pancreatitis if overdosed [45,46]. Adjuvant medication therapy with either Sulfonylureas such as Glucotrol or Meglitinides such as Repaglinide can effectively stimulate pancreas β-cells to secrete more insulin in the short term; however, Sulfonylureas can cause the progressive dysfunction of pancreas β-cells in a long-term treatment. Furthermore, they also aggravate the risk of gaining weight and lowering blood glucose levels [47,48]. Thiazolidinediones is an analogue of Metformin, rendering tissues more sensitive to the insulin. However, it may as well result in overweight and even severe side effects, i.e., heart failure and anemia. DPP-4 inhibitors that prevent DPP-4 from degrading GLP-1, e.g., Sitagliptin, and GLP-1 receptor agonists that affect GLP-1 to last longer, e.g., Liraglutide, are of particular interest for their glucose-lowering effects, which are useful to the treatment of T2D [49]. Although they are at very low risk of hypoglycemia and are also known to help with weight loss, these medications might lead to gastrointestinal disorders, e.g., nausea, diarrhea, or constipation, and overdosage often increases the probability of pancreatitis occurrence [50,51]. SGLT2 inhibitors are one of the newer medications used to lower blood sugar in patients with T2D. Unlike most anti-diabetic drugs that work by either increasing insulin in the body or increasing the insulin sensitivity of cells, SGLT2 inhibitors, e.g., Dapagliflozin, Canagliflozin, Empagliflozin, etc., cause kidneys to excrete glucose into urine to reduce the blood sugar level [52]. However, excess sugar in urine creates a cozy environment for bacteria and fungi to thrive in the urinary tract or genital area, giving rise to urinary tract infection (about 50% greater in patients with diabetes) [53]. In addition, some patients may experience increased frequency of urination, which leads to lower blood pressure due to the loss of fluids; others may notice a slight increase in their cholesterol values [54]. Therefore, discovering effective treatments and promising medications for T2D is still needed.

The proposed drug combination of Sulforaphane and Biotin not only is natural and readily available from daily life but also holds a chance to keep the body from inflammatory-dependent apoptosis and fat accumulation. Although further evaluation in clinical trials is still needed and the potential side effects after consuming should be monitored, the newfound medication indeed brings hope to improve T2D management.

## 4. Materials and Methods

### 4.1. Overview the Procedure of Systems Biology and Systematic Drug Discovery and Design for Type 2 Diabetes (T2D) and Non-T2D

To investigate and gain much more understanding of the T2D pathogenesis, we applied a systems biology approach [55] to build core signaling pathways and explored discrepancies between non-T2D and T2D from the perspective of molecular genetics and epigenetics. Furthermore, a systematic drug discovery procedure was proposed to discover and design a promising drug combination for treating T2D. Notably, drug design specifications were further utilized for screening potential drugs from predicted candidate drugs. The procedure of a systems biology approach and the outline of systematic drug discovery and design method is shown in Figure 1 and subdivided into a few steps:

#### 4.1.1. The Construction of Candidate GWGENs

The candidate protein–protein interaction network (PPIN) and candidate gene regulatory network (GRN) were constructed and integrated into GWGEN for non-T2D and T2D respectively by mining the protein–protein interaction and gene regulation databases.

#### 4.1.2. The Identification of Real GWGENs

The system identification and system order detection method Akaike information criterion (AIC) are used to remove the false positive protein interactions and gene regulations in candidate GWGENs to obtain the real GWGENs via the RNA-seq data downloaded from NCBI GSE81608.

#### 4.1.3. Extracting Core GWGENs by Principal Network Projection (PNP) Method

The core GWGENs were obtained through extracting 85% of the principal network components consisting of the top 3000 proteins, genes, miRNAs, and lncRNAs by the PNP method from the viewpoint of network significance.

#### 4.1.4. The Explorations of Core Signaling Pathways

According to the nodes and edges in core GWGENs and the KEGG pathways annotations, the core signaling pathways were established for T2D and non-T2D. Subsequently, we investigated the genetic and epigenetic pathogenic mechanism by comparing their core signaling pathways.

#### 4.1.5. Potential Multiple-Molecule Targeting Drug Discovery

The deep neural network (DNN) was trained for the drug–target interaction model (DTI) via the drug–target interaction database. With the help of the DTI model, drugs having the possible interactions with biomarkers were predicted to be the candidate drugs. Then, the potential multiple-molecule targeting drug was selected for T2D treatment before clinical trials from the candidate drugs according to the drug design specifications of drug regulation ability, toxicity, sensitivity, and side effect.

### 4.2. Data Mining, Preprocessing and Candidate GWGENs Construction

In our research, the dataset with accession number GSE81608 was downloaded from the gene expression omnibus (GEO) of the National Center for Biotechnology Information (NCBI), and its relevant experimental platform was GPL16791. The dataset contained mRNA expression levels of genes, proteins, miRNAs, TFs, receptors, and lncRNAs in pancreatic α-cell, β-cell, δ-cell, and PP-cell. The samples of the dataset were assorted into two categories, i.e., T2D and non-T2D. In this study, to identify the T2D pathogenic mechanism on the pancreatic β-cell, the samples of the subtype β-cell were specifically extracted from the original experimental data. Furthermore, according to the WHO report, the age distribution of incidence in diabetes is at the range of approximately 50 years old and older. Therefore, 86 and 123 samples were chosen respectively for T2D and non-T2D with age equal to or greater than to 50 years old. Then, we constructed the candidate PPIN based on the Database of Interacting Proteins (DIP) [56], IntAct [57], the Biological General Repository for Interaction Datasets database (BioGRID) [58], the Biomolecular Interaction Network Database (BIND) [59], and the Molecular INTeraction Database (MINT) [60]. In addition, the candidate GRN was built based on the Integrated Transcription Factor Platform database (ITFP) [61], the Human Transcriptional Regulation Interactions database (HTRI) [62], and the TRANScription FACtor database (TRANSFAC) [63]. MiRNAs and lncRNAs regulations in GRN were referenced to the TargetScanHuman database [64], CircuitsDB [65], and StarBase2.0 [66].

### 4.3. Constructing the Systematic Model for the Candidate GWGEN of T2D and Non-T2D

For the purpose of imitating the human cellular system, we built the stochastic interactive and regulatory models to describe the candidate GWGEN. The candidate GWGEN was composed of PPIN containing the protein–protein interactions and GRN containing the regulations of genes, miRNAs, and lncRNAs. Next, we described the interactions of proteins and regulations of genes, lncRNAs, and miRNAs using the protein–protein interactive model (PPIM), gene regulatory model (GRM), lncRNA regulatory model (LRM), and miRNA regulatory model (MRM) in detail.

First, the q-th protein in PPIM can be described as the following equations:

(1) $$p_{q}\left[n\right]=\sum_{\begin{array}{l} r=1\\ r\neq q \end{array}}^{G_{q}}\kappa_{qr}p_{q}\left[n\right]p_{r}\left[n\right]+\lambda_{q,PPIM}+\mu_{q,PPIM}\left[n\right],\;\mathrm{for}\;q=1,\ldots ,Q,\;n=1,\ldots ,N$$

where $p_{q}\left[n\right]$ indicates the expression level of the q-th protein in the n-th sample and $p_{r}\left[n\right]$ indicates the expression level of the r-th protein in the n-th sample; $\kappa_{qr}$ denotes the interaction ability between the q-th protein and the r-th protein; $G_{q}$ represents the total number of proteins that interact with the q-th protein; Q denotes the total number of proteins in candidate PPIM; N means the total number of samples in our data; $\lambda_{q,PPIM}$ shows the basal level in the model of the q-th protein due to unknown protein interactions of histone modifications such as phosphorylation and acetylation; and $\mu_{q,PPIM}\left[n\right]$ expresses the data noise of the q-th protein.

Second, the transcriptional regulation of the x-th gene in GRM is given as below:

(2) $$\begin{array}{l} g_{x}\left[n\right]=\sum_{\begin{array}{l} u=1\\ u\neq x \end{array}}^{U_{x}}\alpha_{xu}t_{u}\left[n\right]+\sum_{v=1}^{V_{x}}\beta_{xv}l_{v}\left[n\right]-\sum_{w=1}^{W_{x}}\gamma_{xw}m_{w}\left[n\right]g_{x}\left[n\right]+\lambda_{x,GRM}+\mu_{x,GRM}\left[n\right]\\ ,\;\mathrm{for}\;x=1,\ldots ,X,\;n=1,\ldots ,N \end{array}$$

where $g_{x}\left[n\right]$ denotes the expression level of the x-th gene in the n-th sample; $t_{u}$[n], $l_{v}$[n], and $m_{w}$[n] individually indicate the expression level of the u-th TF, the v-th lncRNA and the w-th miRNA of the n-th sample; $U_{x}$, $V_{x}$, and $W_{x}$ separately mean the total binding number of TFs, lncRNAs and miRNAs; $\alpha_{xu}$ shows the transcriptional regulatory ability from the u-th TF to the x-th gene; $\beta_{xv}$ represents the transcriptional regulatory ability from the v-th lncRNA to the x-th gene; $\gamma_{xw}$≥0 expresses the post-transcriptional regulatory ability of the w-th miRNA on the x-th gene; X denotes the total number of gene in GRNs; N indicates the total number of data samples; $\lambda_{x,GRM}$ means the basal level of the x-th gene because of the unknown gene regulations such as methylation; and $\mu_{x,GRM}\left[n\right]$ is the data noise.

Third, TFs, lncRNAs, and miRNAs also have a potential impact on the i-th lncRNA and we can depict this behavior by the LRM in candidate GWGENs. The equation is obtained as follows:

(3) $$\begin{array}{l} l_{i}\left[n\right]=\sum_{u=1}^{U_{i}}\sigma_{iu}t_{u}\left[n\right]+\sum_{\begin{array}{l} v=1\\ v\neq i \end{array}}^{V_{i}}\varsigma_{iv}l_{v}\left[n\right]-\sum_{w=1}^{W_{i}}\tau_{iw}m_{w}\left[n\right]l_{i}\left[n\right]+\lambda_{i,LRM}+\mu_{i,LRM}\left[n\right]\\ ,\;\mathrm{for}\;i=1,\ldots ,I,\;n=1,\ldots ,N \end{array}$$

where $l_{i}\left[n\right]$ indicates the expression level of the i-th lncRNA; $t_{u}\left[n\right]$, $l_{v}\left[n\right]$, and $m_{w}\left[n\right]$ represent the expression level of the u-th TF, the v-th lncRNA, and the w-th miRNA of the n-th sample, respectively; $U_{i}$, $V_{i}$, and $W_{i}$ individually show the total binding number of TFs, lncRNAs and miRNAs. $\sigma_{iu}$ expresses the transcriptional regulatory ability from the u-th TF to the i-th lncRNA; $\varsigma_{iv}$ means the transcriptional regulatory ability from the v-th lncRNA to the i-th lncRNA; $\tau_{iw}\geq 0$ denotes the post-transcriptional regulatory ability from the w-th miRNA to the i-th lncRNA; I is the total number of lncRNAs and N indicates the total number of samples; $\lambda_{i,LRM}$ denotes the basal level of the i-th lncRNA; $\mu_{i,LRM}\left[n\right]$ expresses the data noise.

Fourth, the expression of the j-th miRNA is also affected by the TFs, lncRNAs, and miRNAs. Furthermore, we can illustrate MRM in candidate GWGENs through the following equation:

(4) $$\begin{array}{l} m_{j}\left[n\right]=\sum_{u=1}^{U_{j}}\omega_{ju}t_{u}\left[n\right]+\sum_{\begin{array}{l} v=1\\ v\neq j \end{array}}^{V_{j}}\xi_{jv}l_{v}\left[n\right]-\sum_{w=1}^{W_{j}}\psi_{jw}m_{w}\left[n\right]m_{j}\left[n\right]+\lambda_{j,MRM}+\mu_{j,MRM}\left[n\right]\\ ,\;\mathrm{for}\;j=1,\ldots ,J,\;n=1,\ldots ,N \end{array}$$

where $m_{j}\left[n\right]$ means the expression level of j-th miRNA; $t_{u}\left[n\right]$, $l_{v}\left[n\right]$, and $m_{w}\left[n\right]$ separately represent the expression level of the u-th TF, the v-th lncRNA and the w-th miRNA, respectively; $U_{j}$, $V_{j}$, and $W_{j}$ show the binding total number of TFs, lncRNAs and miRNAs; $\omega_{ju}$ denotes the transcriptional regulatory ability from the u-th TF to the j-th miRNA; $\xi_{jv}$ expresses the transcriptional regulatory ability from the v-th lncRNA to the j-th miRNA; $\psi_{jw}$ indicates the post-transcriptional regulatory ability from the w-th miRNA to the j-th miRNA; J is the total number of miRNAs and N indicates the total number of samples; $\lambda_{j.MRM}$ is the basal level of the j-th miRNA; $\mu_{j,MRM}\left[n\right]$ denotes the data noise.

### 4.4. The System Identification and System Order Detection Methods for Real GWGENs Identification

According to the above stochastic models, PPIM in (1) composed the candidate PPIN; GRM in (2), LRM in (3), and MRM in (4) constituted the candidate GRN. We made use of the system identification and system order detection methods to obtain the real GWGENs of T2D and non-T2D by the corresponding RNA-seq data, respectively. In order to identify the parameters of these stochastic models, Equations (1)–(4) could separately be rewritten as the following linear regression forms.

(5) $$p_{q}[n]=\left[\begin{array}{ccccc} p_{q}[n]p_{1}[n] & p_{q}[n]p_{2}[n] & \cdots & p_{q}[n]p_{G_{q}}[n] & 1 \end{array}\right]\times \left[\begin{array}{c} \kappa_{q1}\\ \kappa_{q2}\\ \vdots \\ \kappa_{qG_{q}}\\ \lambda_{q,PPIM} \end{array}\right]+\mu_{q,PPIM}[n]$$

(6) $$g_{x}[n]=\left[\begin{array}{cccccccccc} t_{1}[n] & \cdots & t_{U_{x}} & l_{1}[n] & \cdots & l_{V_{x}} & m_{1}[n]g_{x}\left[n\right] & \cdots & m_{W_{x}}[n]g_{x}\left[n\right] & 1 \end{array}\right]\times \left[\begin{array}{c} \alpha_{x1}\\ \vdots \\ \alpha_{xU_{x}}\\ \beta_{1}\\ \vdots \\ \beta_{xV_{x}}\\ -\gamma_{1}\\ \vdots \\ -\gamma_{xWx}\\ \lambda_{x,GRM} \end{array}\right]+\mu_{x,GRM}[n]$$

(7) $$l_{i}[n]=\left[\begin{array}{cccccccccc} t_{1}[n] & \cdots & t_{U_{i}} & l_{1}[n] & \cdots & l_{V_{i}} & m_{1}[n]l_{i}\left[n\right] & \cdots & m_{W_{i}}[n]l_{i}\left[n\right] & 1 \end{array}\right]\times \left[\begin{array}{c} \alpha_{i1}\\ \vdots \\ \alpha_{iU_{i}}\\ \beta_{1}\\ \vdots \\ \beta_{iV_{i}}\\ -\gamma_{1}\\ \vdots \\ -\gamma_{iW_{i}}\\ \lambda_{i,LRM} \end{array}\right]+\mu_{i,LRM}[n]$$

(8) $$m_{j}[n]=\left[\begin{array}{cccccccccc} t_{1}[n] & \cdots & t_{U_{j}} & l_{1}[n] & \cdots & l_{V_{j}} & m_{1}[n]m_{j}\left[n\right] & \cdots & m_{W_{j}}[n]m_{j}\left[n\right] & 1 \end{array}\right]\times \left[\begin{array}{c} \alpha_{j1}\\ \vdots \\ \alpha_{jU_{j}}\\ \beta_{1}\\ \vdots \\ \beta_{jV_{j}}\\ -\gamma_{1}\\ \vdots \\ -\gamma_{jW_{j}}\\ \lambda_{j,MRM} \end{array}\right]+\mu_{j,MRM}[n]$$

for q=1,…,Q, x=1,…,X, i=1,…,I, j=1,…,J, n=1,…,N, where (5), (6), (7), and (8) are separately regression forms for PPIM, GRM, LRM, and MRM. Q, X, I, and J are respectively the total number of proteins, genes, lncRNAs and miRNAs in the candidate GWGWN, and N is the total number of samples.

The linear regression forms in (5), (6), (7), and (8) could be simplified as the following formulas:

(9) $$p_{q}\left[n\right]=\phi_{q,PPIM}\left[n\right]\cdot \theta_{q,PPIM}+\varepsilon_{q,PPIM},\;\mathrm{for}\;q=1,\ldots ,Q$$

(10) $$g_{x}\left[n\right]=\phi_{x,GRM}\left[n\right]\cdot \theta_{x,GRM}+\varepsilon_{x,GRM},\;\mathrm{for}\;x=1,\ldots ,X$$

(11) $$l_{i}\left[n\right]=\phi_{i,LRM}\left[n\right]\cdot \theta_{i,LRM}+\varepsilon_{i,LRM},\;\mathrm{for}\;i=1,\ldots ,I$$

(12) $$m_{j}\left[n\right]=\phi_{j,MRM}\left[n\right]\cdot \theta_{j,MRM}+\varepsilon_{j,MRM},\;\mathrm{for}\;j=1,\ldots ,J$$

where the $\Phi_{q,PPIM}\left[n\right]$, $\Phi_{x,GRM}\left[n\right]$, $\Phi_{i,LRM}\left[n\right]$, and $\Phi_{j,MRM}\left[n\right]$ individually denote the regression vectors of proteins, gene, lncRNAs, and miRNAs in the n-th sample; $\theta_{q,PPIM}$ means the parameter vector of the protein-protein interaction abilities and protein basal levels; $\theta_{x,GRM}$, $\theta_{i,LRM}$, and $\theta_{j,MRM}$ are the parameter vector of the transcriptional regulatory abilities and basal levels of the genes, lncRNAs, and miRNAs, respectively; $\varepsilon_{q,PPIM}$, $\varepsilon_{x,GRM}$, $\varepsilon_{i,LRM}$, and $\varepsilon_{j,MRM}$ are individually the data noise for PPIM, GRM, LRM and MRM.

For N samples, the above regression equations are given as below:

(13) $$\left[\begin{array}{c} p_{q}\left[1\right]\\ p_{q}\left[2\right]\\ \vdots \\ p_{q}\left[N\right] \end{array}\right]=\left[\begin{array}{c} \phi_{q,PPIM}\left[1\right]\\ \phi_{q,PPIM}\left[2\right]\\ \vdots \\ \phi_{q,PPIM}\left[N\right] \end{array}\right]\cdot \theta_{q,PPIM}+\left[\begin{array}{c} \varepsilon_{q,PPIM}\left[1\right]\\ \varepsilon_{q,PPIM}\left[2\right]\\ \vdots \\ \varepsilon_{q,PPIM}\left[N\right] \end{array}\right],\;\mathrm{for}\;q=1,\ldots ,Q$$

(14) $$\left[\begin{array}{c} g_{x}\left[1\right]\\ g_{x}\left[2\right]\\ \vdots \\ g_{x}\left[N\right] \end{array}\right]=\left[\begin{array}{c} \phi_{x,GRM}\left[1\right]\\ \phi_{x,GRM}\left[2\right]\\ \vdots \\ \phi_{x,GRM}\left[N\right] \end{array}\right]\cdot \theta_{x,GRM}+\left[\begin{array}{c} \varepsilon_{x,GRM}\left[1\right]\\ \varepsilon_{x,GRM}\left[2\right]\\ \vdots \\ \varepsilon_{x,GRM}\left[N\right] \end{array}\right],\;\mathrm{for}\;x=1,\ldots ,X$$

(15) $$\left[\begin{array}{c} l_{i}\left[1\right]\\ l_{i}\left[2\right]\\ \vdots \\ l_{i}\left[N\right] \end{array}\right]=\left[\begin{array}{c} \phi_{i,LRM}\left[1\right]\\ \phi_{i,LRM}\left[2\right]\\ \vdots \\ \phi_{i,LRM}\left[N\right] \end{array}\right]\cdot \theta_{i,LRM}+\left[\begin{array}{c} \varepsilon_{i,LRM}\left[1\right]\\ \varepsilon_{i,LRM}\left[2\right]\\ \vdots \\ \varepsilon_{i,LRM}\left[N\right] \end{array}\right],\;\mathrm{for}\;i=1,\ldots ,I$$

(16) $$\left[\begin{array}{c} m_{j}\left[1\right]\\ m_{j}\left[2\right]\\ \vdots \\ m_{j}\left[N\right] \end{array}\right]=\left[\begin{array}{c} \phi_{j,MRM}\left[1\right]\\ \phi_{j,MRM}\left[2\right]\\ \vdots \\ \phi_{j,MRM}\left[N\right] \end{array}\right]\cdot \theta_{j,MRM}+\left[\begin{array}{c} \varepsilon_{j,MRM}\left[1\right]\\ \varepsilon_{j,MRM}\left[2\right]\\ \vdots \\ \varepsilon_{j,MRM}\left[N\right] \end{array}\right],\;\mathrm{for}\;j=1,\ldots ,J$$

The above equations could be individually represented as the follows:

(17) $$P_{q}=\Phi_{q,PPIM}\cdot \Theta_{q,PPIM}+{Ε}_{q,PPIM},\;\mathrm{for}\;q=1,\ldots ,Q$$

(18) $$G_{x}=\Phi_{x,GRM}\cdot \Theta_{x,GRM}+{Ε}_{x,GRM},\;\mathrm{for}\;x=1,\ldots ,X$$

(19) $$L_{i}=\Phi_{i,LRM}\cdot \Theta_{i,LRM}+{Ε}_{i,LRM},\;\mathrm{for}\;i=1,\ldots ,I$$

(20) $$M_{j}=\Phi_{j,MRM}\cdot \Theta_{j,MRM}+{Ε}_{j,MRM},\;\mathrm{for}\;j=1,\ldots ,J$$

where $\Phi_{q,PPIM}$, $\Phi_{x,GRM}$, $\Phi_{i,LRM}$, and $\Phi_{j,MRM}$ are separately the regression matrix of proteins, genes, lncRNAs and miRNAs of N samples. $\Theta_{q,PPIM}$, $\Theta_{x,GRM}$, $\Theta_{i,LRM}$, and $\Theta_{j,MRM}$ are the corresponding interactive and regulatory parameter vectors. ${Ε}_{q,PPIM}$, ${Ε}_{x,GRM}$, ${Ε}_{i,LRM}$, and ${Ε}_{j,MRM}$ are the corresponding data noise vectors.

What is worth noticing is that the maximum degree of the parameter estimation of proteins in PPIs and genes in GRNs must be less than the samples; otherwise, it would cause the overfitting problem during the process of system identification.

Firstly, for the purpose of identifying the real GWGENs, we adopted the least square method to estimate the parameter vectors $\theta_{q,PPIM}$, $\theta_{q,GRM}$, $\theta_{q,LRM}$, and $\theta_{q,MRM}$ with negative regulation constraint on miRNA as follows:

(21) $${\hat{\Theta }}_{q,PPIM}=\underset{\Theta_{q,PPIM}}{\mathrm{argmin}}\frac{1}{2}{\left\|\Phi_{q,PPIM}\cdot \Theta_{q,PPIM}-P_{q}\right\|}_{2}^{2}$$

(22) $$\begin{array}{l} {\hat{\Theta }}_{x,GRM}=\underset{\Theta_{x,GRM}}{\mathrm{argmin}}\frac{1}{2}{\left\|\Phi_{x,GRM}\cdot \Theta_{x,GRM}-G_{x}\right\|}_{2}^{2}\\ \mathrm{subject}\;\mathrm{to}\;\left[\begin{array}{cccc} \underset{U_{i}}{\underset{︸}{\begin{array}{ccccc} 0 & 0 & \cdots & \cdots & 0\\ 0 & 0 & \cdots & \cdots & 0\\ \vdots & \vdots & \ddots & \ddots & \vdots \\ \vdots & \vdots & \ddots & \ddots & \vdots \\ 0 & 0 & \cdots & \cdots & 0 \end{array}}}| & \underset{V_{i}}{\underset{︸}{\begin{array}{ccccc} 0 & 0 & \cdots & \cdots & 0\\ 0 & 0 & \cdots & \cdots & 0\\ \vdots & \vdots & \ddots & \ddots & \vdots \\ \vdots & \vdots & \ddots & \ddots & \vdots \\ 0 & 0 & \cdots & \cdots & 0 \end{array}}}| & \underset{W_{i}}{\underset{︸}{\begin{array}{ccccc} 1 & 0 & \cdots & \cdots & 0\\ 0 & 1 & \cdots & \cdots & 0\\ \vdots & \vdots & \ddots & \ddots & \vdots \\ 0 & 0 & \ddots & 1 & 0\\ 0 & 0 & \cdots & 0 & 1 \end{array}}}| & \begin{array}{c} 0\\ 0\\ \vdots \\ \vdots \\ 0 \end{array} \end{array}\right]\Theta_{i,GRM}\leq \left[\begin{array}{c} 0\\ 0\\ \vdots \\ \vdots \\ 0 \end{array}\right] \end{array}$$

(23) $$\begin{array}{l} {\hat{\Theta }}_{i,LRM}=\underset{\Theta_{i,LRM}}{\mathrm{argmin}}\frac{1}{2}{\left\|\Phi_{i,LRM}\cdot \Theta_{i,LRM}-L_{i}\right\|}_{2}^{2}\\ \mathrm{subject}\;\mathrm{to}\;\left[\begin{array}{cccc} \underset{U_{i}}{\underset{︸}{\begin{array}{ccccc} 0 & 0 & \cdots & \cdots & 0\\ 0 & 0 & \cdots & \cdots & 0\\ \vdots & \vdots & \ddots & \ddots & \vdots \\ \vdots & \vdots & \ddots & \ddots & \vdots \\ 0 & 0 & \cdots & \cdots & 0 \end{array}}}| & \underset{V_{i}}{\underset{︸}{\begin{array}{ccccc} 0 & 0 & \cdots & \cdots & 0\\ 0 & 0 & \cdots & \cdots & 0\\ \vdots & \vdots & \ddots & \ddots & \vdots \\ \vdots & \vdots & \ddots & \ddots & \vdots \\ 0 & 0 & \cdots & \cdots & 0 \end{array}}}| & \underset{W_{i}}{\underset{︸}{\begin{array}{ccccc} 1 & 0 & \cdots & \cdots & 0\\ 0 & 1 & \cdots & \cdots & 0\\ \vdots & \vdots & \ddots & \ddots & \vdots \\ 0 & 0 & \ddots & 1 & 0\\ 0 & 0 & \cdots & 0 & 1 \end{array}}}| & \begin{array}{c} 0\\ 0\\ \vdots \\ \vdots \\ 0 \end{array} \end{array}\right]\Theta_{i,LRM}\leq \left[\begin{array}{c} 0\\ 0\\ \vdots \\ \vdots \\ 0 \end{array}\right] \end{array}$$

(24) $$\begin{array}{l} {\hat{\Theta }}_{j,MRM}=\underset{\Theta_{j,MRM}}{\mathrm{argmin}}\frac{1}{2}{\left\|\Phi_{j,MRM}\cdot \Theta_{j,MRM}-M_{j}\right\|}_{2}^{2}\\ \mathrm{subject}\;\mathrm{to}\;\left[\begin{array}{cccc} \underset{U_{j}}{\underset{︸}{\begin{array}{ccccc} 0 & 0 & \cdots & \cdots & 0\\ 0 & 0 & \cdots & \cdots & 0\\ \vdots & \vdots & \ddots & \ddots & \vdots \\ \vdots & \vdots & \ddots & \ddots & \vdots \\ 0 & 0 & \cdots & \cdots & 0 \end{array}}}| & \underset{V_{j}}{\underset{︸}{\begin{array}{ccccc} 0 & 0 & \cdots & \cdots & 0\\ 0 & 0 & \cdots & \cdots & 0\\ \vdots & \vdots & \ddots & \ddots & \vdots \\ \vdots & \vdots & \ddots & \ddots & \vdots \\ 0 & 0 & \cdots & \cdots & 0 \end{array}}}| & \underset{W_{j}}{\underset{︸}{\begin{array}{ccccc} 1 & 0 & \cdots & \cdots & 0\\ 0 & 1 & \cdots & \cdots & 0\\ \vdots & \vdots & \ddots & \ddots & \vdots \\ 0 & 0 & \ddots & 1 & 0\\ 0 & 0 & \cdots & 0 & 1 \end{array}}}| & \begin{array}{c} 0\\ 0\\ \vdots \\ \vdots \\ 0 \end{array} \end{array}\right]\Theta_{j,MRM}\leq \left[\begin{array}{c} 0\\ 0\\ \vdots \\ \vdots \\ 0 \end{array}\right] \end{array}$$

Based on the above constrained optimization problems in Equations (21)–(24), we sought out the optimal solution of the interactive ability parameters among proteins ${\hat{\Theta }}_{q,PPIM}$, the regulatory parameters of genes ${\hat{\Theta }}_{x,GRM}$, lncRNAs ${\hat{\Theta }}_{i,LRM}$ and miRNAs ${\hat{\Theta }}_{j,MRM}$ via the RNA-seq data of non-T2D and T2D, respectively. The above optimization problems for parameter estimation could be solved by the MATLAB optimization toolbox. Carefully, the negative inequality constraints in Equations (21)–(24) mean that the regulatory parameters of miRNAs should be less than or equal to zero to ensure the negative regulation of miRNAs on genes, lncRNAs and miRNAs.

After the parameter estimation of candidate GWGENs of non-T2D and T2D by the corresponding RNA-seq data, we used the system order detection method, AIC, to detect the system order (the number of interactions of each protein or the number of regulations of each gene, lncRNA and miRNA). The detailed equations of AIC for each protein, gene, lncRNA and miRNA are shown below.

(25) $$\begin{array}{l} AIC(Q_{q})=\log(\Omega_{q,PPIM})+\frac{2(G_{q}+1)}{N}\\ ,\;\mathrm{for}\;\Omega_{q,PPIM}=\frac{{\left(P_{q}-\Phi_{q,PPIM}\cdot {\hat{\Theta }}_{q,PPIM}\right)}^{T}(P_{q}-\Phi_{q,PPIM}\cdot {\hat{\Theta }}_{q,PPIM})}{N} \end{array}$$

where $\Omega_{q,PPIM}$ means the estimated residual error of the q-th protein for the least square parameter estimation ${\hat{\Theta }}_{q,PPIM}$ in (21) and $Q_{q}$ denotes the number of protein interactions with the q-th protein.

(26) $$\begin{array}{l} AIC(U_{x},V_{x},W_{x})=\log(\Omega_{x,GRM})+\frac{2(O_{x,GRM}+1)}{N}\\ ,\;\mathrm{for}\;\Omega_{x,GRM}=\frac{{\left(G_{x}-\Phi_{x,GRM}\cdot {\hat{\Theta }}_{x,GRM}\right)}^{T}(G_{x}-\Phi_{x,GRM}\cdot {\hat{\Theta }}_{x,GRM})}{N},O_{x,GRM}=U_{x}+V_{x}+W_{x} \end{array}$$

where $\Omega_{x,GRM}$ denotes the estimated residual error of the x-th gene in (22) and $O_{x,GRM}$ means the number of regulations of the genes, lncRNAs and miRNAs on the x-th gene; ${\hat{\Theta }}_{x,GRM}$ is the estimated parameters in (22).

(27) $$\begin{array}{l} AIC(U_{i},V_{i},W_{i})=\log(\Omega_{i,LRM})+\frac{2(O_{i,LRM}+1)}{N}\\ ,\;\mathrm{for}\;\Omega_{i,LRM}=\frac{{\left(L_{i}-\Phi_{i,LRM}\cdot {\hat{\Theta }}_{i,LRM}\right)}^{T}(L_{i}-\Phi_{i,LRM}\cdot {\hat{\Theta }}_{i,LRM})}{N},O_{i,LRM}=U_{i}+V_{i}+W_{i} \end{array}$$

where $\Omega_{i,LRM}$ shows the estimated residual error of the i-th lncRNA in (23) and $O_{i,LRM}$ indicates the number of regulations of the genes, lncRNAs and miRNAs on the i-th lncRNA; ${\hat{\Theta }}_{i,LRM}$ expresses the estimated parameters in (23).

(28) $$\begin{array}{l} AIC(U_{j},V_{j},W_{j})=\log(\Omega_{j,MRM})+\frac{2(O_{j,MRM}+1)}{N}\\ ,\;\mathrm{for}\;\Omega_{j,MRM}=\frac{{\left(M_{j}-\Phi_{j,MRM}\cdot {\hat{\Theta }}_{j,MRM}\right)}^{T}(M_{j}-\Phi_{j,MRM}\cdot {\hat{\Theta }}_{j,MRM})}{N},O_{j,MRM}=U_{j}+V_{j}+W_{j} \end{array}$$

where $\Omega_{j.MRM}$ expresses the estimated residual error of the j-th miRNA in (24) and $O_{j.MRM}$ represents the number of parameters regulations of the genes, lncRNAs and miRNAs on the j-th miRNA; ${\hat{\Theta }}_{j,MRM}$ is the estimated parameter in (24).

According to the order detection of AIC in system identification [67], the real order of a system (i.e., the number of interactions of the q-th protein in (1) or the number of regulations on the x-th in (2)) is to minimize the AIC. Therefore, the true number of interactions or regulations for each protein, gene, lnRNA and miRNA in candidate GWGENs can be obtained by solving the following AIC minimization problems.

(29) $$Q_{q}^{*}=\underset{Q_{q}}{\mathrm{argmin}}AIC(G_{q}),\;\mathrm{for}\;q=1,\ldots ,Q$$

(30) $$U_{x}^{*},V_{x}^{*},W_{x}^{*}=\underset{U_{x},V_{x},W_{x}}{\mathrm{argmin}}AIC(U_{x},V_{x},W_{x}),\;\mathrm{for}\;x=1,\ldots ,X$$

(31) $$U_{i}^{*},V_{i}^{*},W_{i}^{*}=\underset{U_{i},V_{i},W_{i}}{\mathrm{argmin}}AIC(U_{i},V_{i},W_{i}),\;\mathrm{for}\;i=1,\ldots ,I$$

(32) $$U_{j}^{*},V_{j}^{*},W_{j}^{*}=\underset{U_{j},V_{j},W_{j}}{\mathrm{argmin}}AIC(U_{j},V_{j},W_{j}),\;\mathrm{for}\;j=1,\ldots ,J$$

where $Q_{q}^{*}$ denoted the true number of protein interactions for the q-th protein; $U_{x}^{*},V_{x}^{*},W_{x}^{*}$ individually indicate the true number of regulations of genes, lncRNAs and miRNAs on the x-th gene; $U_{i}^{*},V_{i}^{*},W_{i}^{*}$ denote the true number of regulations of genes, lncRNAs and miRNAs on the i-th lncRNA, respectively; $U_{j}^{*},V_{j}^{*},W_{j}^{*}$ are separately the true number of regulations of genes, lncRNAs and miRNAs on the j-th miRNA. Therefore, the protein–protein interactions and gene, miRNA, and lncRNA regulations out of true order by AIC minimization problems in (29)–(32) are considered as false positives in candidate GWGEN of non-T2D and T2D and should be removed one by one to obtain the real GWGEN.

### 4.5. The Principal Network Projection (PNP) Method for the Core GWGENs Extraction from Real GWGENs

The real GWGENs of non-T2D and T2D were compared to investigate the genetic and epigenetic pathogenic molecular mechanism. However, it was still harder to analyze the two larger scale and complicated real GEGENs so that we applied the principal network projection (PNP) method on the basis of the singular value decomposition (SVD) to extract the core GWGENs from the real GWGENs. Before studying the core network extraction in depth, we will start by introducing the real GWGEN network matrix H. Network matrix H consists of interactions among proteins and regulations of the TF-gene, TF-lncRNA, TF-miRNA, lncRNA-gene, lncRNA-lncRNA, lncRNA-miRNA, miRNA-gene, miRNA-lncRNA, and miRNA-miRNA in the real GWGEN as the follows:

(33) $$H=\left[\begin{array}{rrr} h_{protein\Leftrightarrow protein} & 0 & 0\\ h_{TF\Rightarrow \mathrm{gene}} & h_{\ln c\mathrm{RNA}\Rightarrow gene} & h_{miRNA\Rightarrow gene}\\ h_{TF\Rightarrow \ln c\mathrm{RNA}} & h_{\ln cRNA\Rightarrow \ln cRNA} & h_{miRNA\Rightarrow \ln cRNA}\\ h_{TF\Rightarrow \mathrm{mi}\mathrm{RNA}} & h_{\ln cRNA\Rightarrow miRNA} & h_{miRNA\Rightarrow miRNA} \end{array}\right]$$

where $h_{protein\Leftrightarrow protein}$ denotes the sub-matrix of PPI of which the bidirectional arrow at the subscript of the parameter means that the protein interaction is bidirectional; $h_{TF\Rightarrow \mathrm{gene}}$, $h_{TF\Rightarrow \ln cRNA}$, $h_{TF\Rightarrow \mathrm{mi}RNA}$, $h_{\ln cRNA\Rightarrow gene}$, $h_{\ln cRNA\Rightarrow \ln cRNA}$, $h_{\ln cRNA\Rightarrow miRNA}$, $h_{miRNA\Rightarrow gene}$, $h_{miRNA\Rightarrow \ln cRNA}$ and $h_{miRNA\Rightarrow miRNA}$ denote the transcriptional regulatory sub-networks of TFs on genes, lncRNAs, and miRNAs; lncRNAs on genes, lncRNAs, and miRNAs; and miRNAs on genes, lncRNAs, and miRNAs, respectively. The detail components of network matrix H of real GWGENs are given below:

(34) $$H=\left[\begin{array}{cccccccccccccccccc} {\hat{\kappa }}_{11} & {\hat{\kappa }}_{12} & \cdots & {\hat{\kappa }}_{1r} & \cdots & {\hat{\kappa }}_{1Q_{q}} & 0 & 0 & \cdots & 0 & \cdots & 0 & 0 & 0 & \cdots & 0 & \cdots & 0\\ {\hat{\kappa }}_{21} & {\hat{\kappa }}_{22} & \cdots & {\hat{\kappa }}_{2r} & \cdots & {\hat{\kappa }}_{2Q_{q}} & 0 & 0 & \cdots & 0 & \cdots & 0 & 0 & 0 & \cdots & 0 & \cdots & 0\\ \vdots & \vdots & \ddots & \vdots & \ddots & \vdots & \vdots & \vdots & \ddots & \vdots & \ddots & \vdots & \vdots & \vdots & \ddots & \vdots & \ddots & \vdots \\ {\hat{\kappa }}_{q1} & {\hat{\kappa }}_{q2} & \cdots & {\hat{\kappa }}_{qr} & \cdots & {\hat{\kappa }}_{qQ_{q}} & 0 & 0 & \cdots & 0 & \cdots & 0 & 0 & 0 & \cdots & 0 & \cdots & 0\\ \vdots & \vdots & \ddots & \vdots & \ddots & \vdots & \vdots & \vdots & \ddots & \vdots & \ddots & \vdots & \vdots & \vdots & \ddots & \vdots & \ddots & \vdots \\ {\hat{\kappa }}_{Q1} & {\hat{\kappa }}_{Q1} & \cdots & {\hat{\kappa }}_{Q1} & \cdots & {\hat{\kappa }}_{QQ_{q}} & 0 & 0 & \cdots & 0 & \cdots & 0 & 0 & 0 & \cdots & 0 & \cdots & 0\\ {\hat{\alpha }}_{11} & {\hat{\alpha }}_{12} & \cdots & {\hat{\alpha }}_{1u} & \cdots & {\hat{\alpha }}_{1U_{x}} & {\hat{\beta }}_{11} & {\hat{\beta }}_{12} & \cdots & {\hat{\beta }}_{1v} & \cdots & {\hat{\beta }}_{1V_{x}} & {\hat{\gamma }}_{11} & {\hat{\gamma }}_{12} & \cdots & {\hat{\gamma }}_{1w} & \cdots & {\hat{\gamma }}_{1W_{x}}\\ {\hat{\alpha }}_{21} & {\hat{\alpha }}_{22} & \cdots & {\hat{\alpha }}_{2u} & \cdots & {\hat{\alpha }}_{2U_{x}} & {\hat{\beta }}_{21} & {\hat{\beta }}_{22} & \cdots & {\hat{\beta }}_{2v} & \cdots & {\hat{\beta }}_{2V_{x}} & {\hat{\omega }}_{21} & {\hat{\omega }}_{22} & \cdots & {\hat{\gamma }}_{2w} & \cdots & {\hat{\gamma }}_{2W_{x}}\\ \vdots & \vdots & \ddots & \vdots & \ddots & \vdots & \vdots & \vdots & \ddots & \vdots & \ddots & \vdots & \vdots & \vdots & \ddots & \vdots & \ddots & \vdots \\ {\hat{\alpha }}_{x1} & {\hat{\alpha }}_{x2} & \cdots & {\hat{\alpha }}_{xu} & \cdots & {\hat{\alpha }}_{xU_{x}} & {\hat{\beta }}_{x1} & {\hat{\beta }}_{x2} & \cdots & {\hat{\beta }}_{xv} & \cdots & {\hat{\beta }}_{xV_{x}} & {\hat{\gamma }}_{x1} & {\hat{\gamma }}_{x2} & \cdots & {\hat{\gamma }}_{xw} & \cdots & {\hat{\gamma }}_{xW_{x}}\\ \vdots & \vdots & \ddots & \vdots & \ddots & \vdots & \vdots & \vdots & \ddots & \vdots & \ddots & \vdots & \vdots & \vdots & \ddots & \vdots & \ddots & \vdots \\ {\hat{\alpha }}_{X1} & {\hat{\alpha }}_{X2} & \cdots & {\hat{\alpha }}_{Xu} & \cdots & {\hat{\alpha }}_{XU_{x}} & {\hat{\beta }}_{X1} & {\hat{\beta }}_{X2} & \cdots & {\hat{\beta }}_{Xv} & \cdots & {\hat{\beta }}_{XV_{x}} & {\hat{\gamma }}_{X1} & {\hat{\gamma }}_{X2} & \cdots & {\hat{\gamma }}_{Xw} & \cdots & {\hat{\gamma }}_{XW_{x}}\\ {\hat{\sigma }}_{11} & {\hat{\sigma }}_{12} & \cdots & {\hat{\sigma }}_{1u} & \cdots & {\hat{\sigma }}_{1U_{i}} & {\hat{\varsigma }}_{11} & {\hat{\varsigma }}_{12} & \cdots & {\hat{\varsigma }}_{1v} & \cdots & {\hat{\varsigma }}_{1V_{i}} & {\hat{\tau }}_{11} & {\hat{\tau }}_{12} & \cdots & {\hat{\tau }}_{1w} & \cdots & {\hat{\tau }}_{1W_{i}}\\ {\hat{\sigma }}_{21} & {\hat{\sigma }}_{22} & \cdots & {\hat{\sigma }}_{2u} & \cdots & {\hat{\sigma }}_{2U_{i}} & {\hat{\varsigma }}_{21} & {\hat{\varsigma }}_{22} & \cdots & {\hat{\varsigma }}_{2v} & \cdots & {\hat{\varsigma }}_{2V_{i}} & {\hat{\tau }}_{21} & {\hat{\tau }}_{22} & \cdots & {\hat{\tau }}_{2w} & \cdots & {\hat{\tau }}_{2W_{i}}\\ \vdots & \vdots & \ddots & \vdots & \ddots & \vdots & \vdots & \vdots & \ddots & \vdots & \ddots & \vdots & \vdots & \vdots & \ddots & \vdots & \ddots & \vdots \\ {\hat{\sigma }}_{i1} & {\hat{\sigma }}_{i2} & \cdots & {\hat{\sigma }}_{iu} & \cdots & {\hat{\sigma }}_{iU_{i}} & {\hat{\varsigma }}_{i1} & {\hat{\varsigma }}_{i2} & \cdots & {\hat{\varsigma }}_{iv} & \cdots & {\hat{\varsigma }}_{iV_{i}} & {\hat{\tau }}_{i1} & {\hat{\tau }}_{i2} & \cdots & {\hat{\tau }}_{iw} & \cdots & {\hat{\tau }}_{iW_{i}}\\ \vdots & \vdots & \ddots & \vdots & \ddots & \vdots & \vdots & \vdots & \ddots & \vdots & \ddots & \vdots & \vdots & \vdots & \ddots & \vdots & \ddots & \vdots \\ {\hat{\sigma }}_{I1} & {\hat{\sigma }}_{I2} & \cdots & {\hat{\sigma }}_{Iu} & \cdots & {\hat{\sigma }}_{IU_{i}} & {\hat{\varsigma }}_{I1} & {\hat{\varsigma }}_{I2} & \cdots & {\hat{\varsigma }}_{Iv} & \cdots & {\hat{\varsigma }}_{IV_{i}} & {\hat{\tau }}_{I1} & {\hat{\tau }}_{I2} & \cdots & {\hat{\tau }}_{Iw} & \cdots & {\hat{\tau }}_{IW_{i}}\\ {\hat{\omega }}_{11} & {\hat{\omega }}_{12} & \cdots & {\hat{\omega }}_{1u} & \cdots & {\hat{\omega }}_{1U_{j}} & {\hat{\xi }}_{11} & {\hat{\xi }}_{12} & \cdots & {\hat{\xi }}_{1v} & \cdots & {\hat{\xi }}_{1U_{j}} & {\hat{\psi }}_{11} & {\hat{\psi }}_{12} & \cdots & {\hat{\psi }}_{1w} & \cdots & {\hat{\psi }}_{1W_{j}}\\ {\hat{\omega }}_{21} & {\hat{\omega }}_{22} & \cdots & {\hat{\omega }}_{2u} & \cdots & {\hat{\omega }}_{2U_{j}} & {\hat{\xi }}_{21} & {\hat{\xi }}_{22} & \cdots & {\hat{\xi }}_{2v} & \cdots & {\hat{\xi }}_{2U_{j}} & {\hat{\psi }}_{21} & {\hat{\psi }}_{22} & \cdots & {\hat{\psi }}_{2w} & \cdots & {\hat{\psi }}_{2W_{j}}\\ \vdots & \vdots & \ddots & \vdots & \ddots & \vdots & \vdots & \vdots & \ddots & \vdots & \ddots & \vdots & \vdots & \vdots & \ddots & \vdots & \ddots & \vdots \\ {\hat{\omega }}_{j1} & {\hat{\omega }}_{j2} & \cdots & {\hat{\omega }}_{ju} & \cdots & {\hat{\omega }}_{jU_{j}} & {\hat{\xi }}_{j1} & {\hat{\xi }}_{j2} & \cdots & {\hat{\xi }}_{jv} & \cdots & {\hat{\xi }}_{jU_{j}} & {\hat{\psi }}_{j1} & {\hat{\psi }}_{j2} & \cdots & {\hat{\psi }}_{jw} & \cdots & {\hat{\psi }}_{jW_{j}}\\ \vdots & \vdots & \ddots & \vdots & \ddots & \vdots & \vdots & \vdots & \ddots & \vdots & \ddots & \vdots & \vdots & \vdots & \ddots & \vdots & \ddots & \vdots \\ {\hat{\omega }}_{J1} & {\hat{\omega }}_{J2} & \cdots & {\hat{\omega }}_{Ju} & \cdots & {\hat{\omega }}_{JU_{j}} & {\hat{\xi }}_{J1} & {\hat{\xi }}_{J2} & \cdots & {\hat{\xi }}_{Jv} & \cdots & {\hat{\xi }}_{JU_{j}} & {\hat{\psi }}_{J1} & {\hat{\psi }}_{J2} & \cdots & {\hat{\psi }}_{Jw} & \cdots & {\hat{\psi }}_{JW_{j}} \end{array}\right]\in {\mathbb{R}}^{\left(Q^{*}+X^{*}+I^{*}+J^{*})\times (U^{*}+V^{*}+W^{*}\right)}$$

where ${\hat{\kappa }}_{qr}$ is the interaction ability of between the q-th protein and the r-th protein; ${\hat{\alpha }}_{xu}$, ${\hat{\beta }}_{xv}$, and ${\hat{\gamma }}_{xw}$ are individually the regulation abilities of the u-th TF on the x-th gene, the v-th lncRNA on the x-th gene, and the w-th miRNA on the x-th gene; ${\hat{\sigma }}_{iu}$, ${\hat{\varsigma }}_{iv}$, and ${\hat{\tau }}_{iw}$ represent the regulation abilities of the u-th TF on the i-th lncRNA, the v-th lncRNA on the i-th lncRNA, and the w-th miRNA on the i-th lncRNA, respectively; ${\hat{\omega }}_{ju}$, ${\hat{\xi }}_{jv}$, and ${\hat{\psi }}_{jw}$ separately show the regulation abilities of the u-th TF on the j-th miRNA, the v-th lncRNA on the j-th miRNA, and the w-th miRNA on the w-th miRNA. In addition, some zeros are omitted in the matrix, which means that there is neither interaction nor regulation between the source and target.

Thereafter, the core GWGENs were obtained by applying PNP on the network matrix H with an energy threshold of 85%. First, the network matrix H is decomposed by singular value decomposition (SVD) as follows [68]:

(35) $$H=SVD^{T}$$

where $S\in {\mathbb{R}}^{\left(Q^{*}+X^{*}+I^{*}+J^{*})\times (Q^{*}+X^{*}+I^{*}+J^{*}\right)}$ and $D^{T}\in {\mathbb{R}}^{\left(U^{*}+V^{*}+W^{*})\times (U^{*}+V^{*}+W^{*}\right)}$ are the unitary singular matrices; $V=diag(v_{1},\cdots ,v_{ii},\cdots v_{U^{*}+V^{*}+W^{*}})\in {\mathbb{R}}^{\left(Q^{*}+X^{*}+I^{*}+J^{*})\times (U^{*}+V^{*}+W^{*}\right)}$ denotes the diagonal matrix of which the components at the diagonal are the singular values of H and are arranged in descending order, i.e., $v_{1}\geq v_{2}\geq \cdots \geq v_{i}\geq \cdots \geq v_{U^{*}+V^{*}+W^{*}}\geq 0$.

(36) $$V=\left[\begin{array}{cccccc} v_{1} & 0 & \cdots & 0 & \cdots & 0\\ 0 & v_{2} & \cdots & 0 & \cdots & 0\\ \vdots & \vdots & \ddots & \vdots & \ddots & \vdots \\ 0 & 0 & \cdots & v_{i} & \cdots & 0\\ \vdots & \vdots & \ddots & \vdots & \ddots & \vdots \\ 0 & 0 & \cdots & 0 & \cdots & v_{U^{*}+V^{*}+W^{*}}\\ 0 & 0 & \cdots & 0 & \cdots & 0\\ \vdots & \vdots & \ddots & \vdots & \ddots & \vdots \\ 0 & 0 & \cdots & 0 & \cdots & 0 \end{array}\right]$$

In addition, we defined the normalization of singular values in (36) as below.

(37) $$E_{i}=\frac{v_{i}^{2}}{\sum_{i=1}^{U^{*}+V^{*}+W^{*}}v_{i}^{2}}\;\mathrm{and}\;\sum_{i=1}^{U^{*}+V^{*}+W^{*}}E_{i}=1$$

(38) $$\sum_{i=1}^{I}E_{i}\geq 0.85$$

From the above formula, the top I significant singular vector structures were selected to represent the system with energy equal to or more than 85%. Then we respectively projected each node of the real GWGEN (i.e., each row of network matrix H) to the top I singular vectors as follows.

(39) $$Z(a,b)=h_{a,:}\cdot d_{b,:}^{T},\;\mathrm{for}\;a=1,\ldots ,Q^{*}+X^{*}+I^{*}+J^{*},\;b=1,\ldots ,I$$

where Z(a,b) denotes the projection value of the a-th node on the b-th significant singular vector; $h_{a,:}$ means the a-th row vector of network matrix H, and $d_{:,b}^{T}$ denotes the the b-th column of $D^{T}$. Next, we define the 2-norm projection value to each node such as protein, gene, lnRNA and miRNA in real GWGEN from the top I significant singular vectors as below.

(40) $$S(a)=\sqrt{\sum_{i=1}^{I}Z^{2}(a,b)},\;\mathrm{for}\;a=1,\ldots ,Q^{*}+X^{*}+I^{*}+J^{*}$$

According to the equation in (40), the top 3000 pivotal proteins, genes, miRNAs, and lncRNAs with higher projection value were selected to construct the core GWGENs for T2D and non-T2D, respectively. Afterwards, the core GWGENs were uploaded to the DAVID website for KEGG pathway enrichment analysis, and the construction of core signaling pathways for non-T2D and T2D were accomplished with the help of the annotation of KEGG pathways. The enrichment analysis was used to validate that our results were associated with T2D. Eventually, the potential biomarkers were chosen through investigating the T2D pathogenesis by comparing the non-T2D and T2D core signaling pathways.

### 4.6. Systematic Drug Discovery Based on Drug Design Specifications for T2D

Based on drug design specifications, we aimed to discover a potential multiple-molecule targeting drug for the identified biomarkers. We proposed a DTI model based on a deep neural network to predict the drug–target interaction between the available drugs and targets (biomarkers). Since it is not enough to consider the drug–target interaction alone for drug design, some specifications, i.e., regulation ability, toxicity, sensitivity and side effect are necessary to sieve the candidate drugs predicted by the DTI model. Then, with these considerations, we suggested an appropriate multiple-molecule targeting drug for T2D treatment before clinical trials.

First, based on the flowchart of the systematic drug discovery method in Figure 3, we accessed an integrated collection of protein–ligand affinity data through BindingDB’s unified interface [69], which harvests the selected data and information from multiple existing databases, i.e., PubChem, ChEMBL, UniProt, DrugBank, etc. (for more details, readers can refer to Appendix B). Recently, the feature-based method, for instance, molecular descriptor, is broadly used to describe the structural and chemical properties of molecules such as characteristics from the 2D and 3D spectrum of structure, molecular weight, hydrophilic, hydrophobicity, etc. It was validated that the chemical properties of the drug and genomic sequence of the target could be described with the molecular descriptor for the purpose of convenient analysis in drug design, since the molecular descriptor can transform complicated chemical properties into a simple numerical feature vector [70,71]. On this ground, we utilized the functions from python package pyBioMed to transform both the drug and target into a descriptor as their features individually under the python2.7 environment. The considered drug features of a molecule included constitutional descriptors, connectivity indices, E-state indices, charge descriptors, molecular properties and kappa shape indices. For the target features, the structural and physicochemical features of proteins and peptides from amino acid sequence such as amino acid composition, dipeptide composition…, etc. are calculated (for more detailed information about the descriptor transformation, readers could access the documents of pyBioMed [72]). Then, the descriptor of the drug and target were combined into a feature vector *v*_drug-target_ corresponding to the drug–target pair as below [73]:

(41) $$v_{\mathrm{drug}-\mathrm{target}}=[D,T]=[d_{1},d_{2},\cdots ,d_{M},t_{1},t_{2},\cdots t_{N}]$$

Among $v_{\mathrm{drug}-\mathrm{target}}$, 363 features for a drug and 996 features for a target were collected, where the former features in *v*_drug-target_ are for the drug and the latter are for the target. $d_{1}$ represents the first drug feature; $t_{1}$ indicates the first target feature; M is the total number of drug features; and N denotes the total number of target features. Before training the DNN-based DTI model, we encountered a problem that features are not in the same standing. Since the variables of the features are measured at different scales, they do not contribute equally to the model fitting and might end up creating a bias, i.e., the feature with a larger value would dominate the result. To deal with this potential problem, a feature-wise scaling is usually implemented prior to model fitting. As powerful techniques of feature scaling, Min-max Normalization and Standardization methods are commonly used for bringing every feature in the same footing without any upfront importance. Although Min-max Normalization can also normalize the data into the same scale, it is much more sensitive to outliers compared to Standardization. Therefore, Standardization was performed on the features before applying principal component analysis (PCA) to improve the model performance, and the corresponding mathematical formulation is shown as follows:

(42) $$d_{i}^{*}=\frac{d_{i}-\mu_{i}}{\sigma_{i}},\;\forall i=1,\ldots ,M$$

(43) $$t_{j}^{*}=\frac{t_{j}-\mu_{j}}{\sigma_{j}},\;\forall j=1,\ldots ,N$$

where $d_{i}$ is the i-th drug feature and the $d_{i}^{*}$ is the i-th drug feature after Standardization; $\mu_{i}$ and $\sigma_{i}$ individually represent the mean and standard deviation of the i-th drug feature; $t_{j}$ indicates the j-th feature of the target and $t_{j}^{*}$ denotes the j-th feature of the target after Standardization; $\mu_{j}$ and $\sigma_{j}$ separately signify the mean and standard deviation of the j-th target feature; M denotes the total number of drug features; and N is the total number of target features.

(44) h=σ(wx+b)

where x and h denote input and output, respectively; w is the weighting matrix and b is the bias vector; σ(·) indicates the activation function with Rectified Linear Unit (ReLU) in the hidden layer and Sigmoid in the output layer. Since the binary classification issue is concerned, the binary-cross entropy is chosen as the cost function to calculate the model loss:

(45) $$C_{n}(w,\;b)=-[{\hat{p}}_{n}\log p_{n}+(1-{\hat{p}}_{n})log(1-p_{n})]$$

(46) $$L(w,\;b)=\;\frac{1}{N}\sum_{n=1}^{N}C_{n}(w,\;b)$$

where $p_{n}$ means the truth label of positive interaction; ${\hat{p}}_{n}$ indicates the predictive probability of positive interaction, $1-p_{n}$ shows the truth label of negative interaction, and $1-{\hat{p}}_{n}$ represents the predicted probability of negative interaction. $L({\hat{p}}_{n},p_{n})$ denotes the average of total loss $C({\hat{p}}_{n},p_{n})$. According to the cost function, the backward propagation algorithm is applied to update the model parameter set θ containing the weighting matrix and bias vector through calculating the gradient of cost function in (46) to get the result in (50) and eventually derive the optimal solution $\theta^{*}$ in (48) as follows.

(47) $$\theta =\left[\begin{array}{c} w\\ b \end{array}\right]$$

(48) $$\theta^{*}=\underset{\theta }{\mathrm{argmin}}L(\theta )$$

(49) $$\theta^{l}=\theta^{l-1}-\eta \nabla L(\theta^{l-1})$$

where l is the l-th epoch of learning procedure; η is the learning rate; and $\nabla L(\theta^{l-1})$ is the gradient of $L(\theta^{l-1})$ as below:

(50) $$\nabla L(\theta^{l-1})=\left[\begin{array}{c} \frac{\partial L(\theta^{l-1})}{\partial w}\\ \frac{\partial L(\theta^{l-1})}{\partial b} \end{array}\right]$$

Based on the backward propagation method, the DNN-based DTI model could adjust the parameters to fit the drug–target interaction data at each iteration well. In addition, the hyperparameters were tuned to not only lower the training time but also achieve the best model performance. We used Adam [74] as an optimizer with a default setting and set the learning rate as 0.0001 to make the model parameter θ converge faster and precisely. We set 100 for epochs and 100 for batch size. For the data, we split one-fourth of the data as testing data and three-fourths of it as training data. Moreover, we further divided the training data into ten equal folds to perform ten-fold cross-validation, in which nine-tenths of them were used for model training and one-tenth was used for validation. Such application is exploited to supervise whether the model was better than that of the former epoch and to guarantee the model stability. Furthermore, to avoid overfitting, not only did we embed the dropout layer (dropout rate = 0.4) behind each hidden layer but also applied the early stopping strategy to monitor whether the test accuracy decreased with the continuous improvement of training accuracy or not. After accomplishing model training as shown in Figure 4, we adopted the AUC (area under the curve) score and ROC (receiver operating characteristics) curve [75] in Figure 7 as the performance measurement. It is one of the most useful evaluation metrics to visualize the model performance when it comes to the binary classification problems. The higher AUC score is that in which the area under the line is larger; the better accuracy is for the DNN-based DTI model predicting the true positive and true negative drug–target interaction. The formulas for the AUC score and ROC curve are shown below.

(51) $$\mathrm{TPR}(\mathrm{True}\;\mathrm{Positive}\;\mathrm{Rate})\;=\;\frac{\mathrm{TP}}{\mathrm{TP}+\mathrm{FN}}$$

(52) $$\mathrm{specificity}=\frac{\mathrm{TN}}{\mathrm{TN}+\mathrm{FP}}$$

(53) $$\mathrm{FPR}(\mathrm{False}\;\mathrm{Positive}\;\mathrm{Rate})\;=\;1-\mathrm{specificity}\;=\;\frac{\mathrm{FP}}{\mathrm{TN}+\mathrm{FP}}$$

where TP (True Positive) means that the real value is true and is judged correctly; TN (True Negative) shows that the real value is true and is judged by mistake; FP (False Positive) indicates the real value is false and is judged accurately; FN (False Negative) represents that the real value is false and is judged in error.

It is worth noting that the majority of previous network approaches use machine learning (ML)-based methods to perform predictions over the drug–target interaction space [76,77,78]. However, such techniques have major limitations. Traditional ML is a time-consuming process and requires lots of expertise to design and run the algorithms. Without a good understanding of the domain knowledge and feature engineering, a traditional machine algorithm can hardly work well.

As a kind of ML-based model with multiple hidden layers and a more complicated parameter training procedure, the deep learning method attracts lots of attention for its relatively better performance and ability to learn representations of data with multiple levels of abstraction [79]. When there is a lack of domain understanding for feature introspection, deep learning techniques outshine others as we do not have to worry much about feature engineering. Additionally, the comparison of deep-learning methods with other acceptable ML algorithms in the task of new DTIs identification has previously been performed as well, where framework based on deep learning could indeed achieve relatively high prediction performance [80]. As a result, for each algorithm compared in our work, only default parameters without fine-tuning were set to learn features from the data. However, a disadvantage should be solved that there are no experimental validated noninteracting drug–target pairs so that it is difficult to select negative samples, which would largely influence the predictive accuracy of the method [81]. Hence, apart from extracting a great number of samples from the presently largest database, BingdingDB, we further followed the criteria in Appendix B to abstract negative examples from existing drug–target interactions, which enabled us to evaluate and manipulate the data more realistically to achieve better performance.

## 5. Conclusions

In this study, on the basis of our proposed combination of systems biology and systematic drug discovery design, we not only investigated the complicated pathogenic molecular mechanism of T2D from genetic and epigenetic perspectives but also discovered a potential drug combination for the clinical treatment of T2D based on four drug design specifications. At first, we constructed the stochastic biological networks by systematic identification and system order detection methods by exploring big data. After that, we extracted the core signaling pathways by the PNP method and the annotation of KEGG pathways to select the significant biomarkers from the pathogenesis of T2D. For the purpose of discovering candidate drugs interacting with these biomarkers, we trained a DNN-based DTI model to predict the possible drug–target interactions. Moreover, we considered the drug regulation ability, toxicity, sensitivity and side effects as the drug design specifications to better sieve appropriate potential drugs. As a result, a set of combinational multiple molecular drugs is proposed as a multiple-molecule targeting drug for T2D treatment. Since the beginning of this century, the advent of the genomic era has presented researchers with a myriad of high throughput genome-wide biological data, which can assist in the interpretation of the indecipherable genetic and epigenetic regulations and the optimization of drug efficacy. Considering the combination of multiple types of genomics data could benefit us to gain deeper insight into the pathogenic mechanism of diseases. It is expected that our systems biology and systematic drug discovery design might provide a new orientation for T2D therapeutics.

## Figures and Tables

**Table 1:** Samples of RNA-seq data on pancreatic β-cells from GSE81608 were selected according to age greater than or equal to 50 years and classified into non-T2D and T2D.

RNA-seq Data	Non-T2D	T2D
Age ≥ 50	86	123

**Table 2:** KEGG pathway enrichment analysis of core T2D signaling pathways using DAVID tool.

KEGG Pathway Enrichment Analysis of T2D Core Signaling Pathways		
Pathway	Gene Number	*p*-Value
mTOR signaling pathway	15	3.1 × 10^−3^
Insulin resistance	22	5.8 × 10^−3^
Regulation of lipolysis in adipocytes	14	6.2 × 10^−3^
PI3K–Akt signaling pathway	50	3.1 × 10^−2^
Type II diabetes mellitus	11	3.2 × 10^−2^

**Table 3:** KEGG pathway enrichment analysis of core non-T2D signaling pathways using DAVID tool.

KEGG Pathway Enrichment Analysis of non-T2D Core Signaling Pathways		
Pathway	Gene Number	*p*-Value
Jak–STAT signaling pathway	25	3.1 × 10^−2^
Cell cycle	22	3.3 × 10^−2^
mTOR signaling pathway	12	5.4 × 10^−2^
p53 signaling pathway	13	6.6 × 10^−2^
AMPK signaling pathway	20	8.9 × 10^−2^

**Table 4:** 10-fold cross-validation measure for the DNN-based DTI model.

Model Performance (10-Fold Cross-Validation)				
	Validation Loss	Validation Accuracy (%)	Testing Loss	Testing Accuracy (%)
1	0.148	95.23	0.159	94.87
2	0.151	94.93	0.150	95.05
3	0.159	94.58	0.155	94.69
4	0.155	94.73	0.161	94.68
5	0.154	94.75	0.156	94.91
6	0.147	94.91	0.155	94.95
7	0.164	94.74	0.157	94.96
8	0.162	94.56	0.158	94.82
**9**	**0.151**	**95.18**	**0.155**	**95.06**
10	0.142	95.2	0.153	94.94
Average	0.153	94.88	0.156	94.89
Standard deviation	0.007	0.252	0.003	0.131

The far-left column recorded the numbers of 10-fold cross-validation models. The block with values in bold denotes the model with best testing accuracy in contrast to the other models and is chosen as the well-trained DTI model for drug–target binding prediction.

**Table 5:** The side effect for candidate drugs on core signaling pathways.

Candidate Drugs	Binding Biomarkers	Binding Numbers Except Desired Target Biomarkers in Core Signaling Pathways
Anisomycin	IKK, STAT3, PPARγ, ETS1, FAS	37
* Sulforaphane	IKK, STAT3, PPARγ, ETS1, FAS	23
Memantine	IKK, STAT3	11
Trimetozine	IKK, STAT3, PPARγ	14
* Biotin	IKK, STAT3, PPARγ, ETS1	19
Gabexate	IKK, STAT3, PPARγ, ETS1	31
Famotidine	IKK, STAT3, PPARγ, ETS1	25
Cilostazol	IKK, STAT3, PPARγ, ETS1	26
Acetylcysteine	IKK, STAT3, PPARγ, ETS1, FAS	41

The side effect of a drug is defined as the number of targets except the desired biomarkers. The candidate drugs with ‘*’ are selected as our potential drugs for T2D.

**Table 6:** The candidate drugs for T2D and their corresponding information.

Candidate Drugs	Regulation Ability to Specific Biomarkers					Toxicity (LD50, mol/kg)	Sensitivity (EC50)
	IKK	STAT3	PPARγ	ETS1	FAS		
Anisomycin	**0.822**	0.809	3.622	**5.507**	−0.184	3.535	−1.099
* Sulforaphane	−0.029	0.079	0.075	**0.089**	−0.059	3.110	−0.008
Memantine	−0.997	0.707				2.346	−0.383
Trimetozine	−0.724	0.650	0.489			2.148	−0.851
* Biotin	−1.214	1.075	0.969	−0.986		2.058	−0.249
Gabexate	−1.324	**−0.942**	1.237	−2.151		1.999	−0.229
Famotidine	−0.693	0.356	**−1.004**	−0.119		1.952	−0.548
Cilostazol	−0.570	**−1.622**	0.387	−1.222		1.889	−0.141
Acetylcysteine	−0.788	0.645	**−0.620**	**1.923**	−1.000	1.294	−0.554

Some of the candidate drugs are denoted and ranked based on their toxicity. The regulation ability block without values represented that no binding between the drug and target existed. The blocks with values in bold indicate unwanted regulations. The positive value of regulation ability signifies the positive regulation, whereas the negative value denotes the downregulation. For each drug, the larger LD50 value it possesses, the lower toxicity it has; the smaller LD50 value it owns, the higher efficacy (sensitivity) it holds. The candidate drugs with ‘*’ are selected as the potential drugs.

**Table 7:** The drug design specifications of a potential multiple-molecule targeting drug for T2D.

Drug Names	Regulation Ability to Specific Biomarkers					Toxicity (LD50, mol/kg)	Sensitivity (EC50)
	IKK	STAT3	PPARγ	ETS1	FAS		
Sulforaphane	✓	✓	✓		✓	3.110	−0.008
Biotin	✓	✓	✓	✓		2.058	−0.249
Sulforaphane				Biotin			
**Binding numbers except their desired target biomarkers in core signaling pathways (side effect)**							
23				19			
**Chemical structures of multiple molecular drugs**							
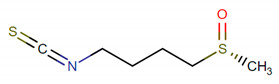				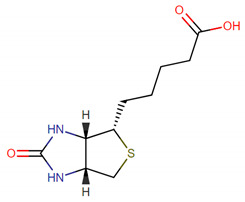			

‘✓’ denotes the drug could bind to the biomarkers with a desired regulation capacity. Among the chemical structures of the multiple molecular drugs, “R(CH2)_n_H” indicates the alkyl group; “RNCS” means the isothiocyanate group; “RSOR′” represents the sulfinyl group; “R′R”NH” is the secondary amine; “RCOR′” means the carbonyl group; ‘RSR′’ is the sulfide group; and the “RCOOH” is the carboxyl group. 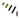 represents a solid wedge where the bond is pointing out toward the viewer and 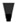 indicates a hashed wedge where the bond is receding away from the viewer.

## Abbreviations

T2D	Type 2 Diabetes
GRN	Gene Regulatory Network
PPIN	Protein-Protein Interaction Network
GWGEN	Genome Wide Genetic and Epigenetic Network
PNP	Principal Network Projection
DTI	Drug–Target Interaction

## Appendix A

**Table A1:** The statistics of the nodes and edges in candidate GWGEN, non-T2D GWGEN, and T2D GWGEN after identification.

	Candidate GWGEN	Non-T2D GWGEN	T2D GWGEN
LncRNA-TF	158	1	4
LncRNA-Receptor	2	2	0
LncRNA-Protein	142	8	3
LncRNAs	384	121	125
MiRNA-TF	17,052	1	3
MiRNA-Receptor	13,438	3	2
MiRNA-Protein	75,629	8	16
MiRNAs	417	22	29
TF-LncRNA	417	133	162
TF-MiRNA	723	25	31
TF-TF	33,897	2249	1984
TF-Receptor	16,241	1059	1084
TF-Protein	84,634	8336	8887
TFs	4351	1086	1057
Receptor-LncRNA	101	29	35
Receptor-MiRNA	78	2	1
Receptor-TF	2520	356	327
Receptor-Receptor	1757	231	183
Receptor-Protein	9111	1991	1737
Receptors	2768	2093	2033
Proteins	19,041	18,101	17,924
PPIs	6,244,695	826,916	814,993
Total nodes	26,961	21,423	21,168
Total edges	6,500,938	841,350	829,452

The content in the table shows the number of nodes or edges. The rows of the table contain different types of node and edge, e.g., LncRNA-TF indicates that lncRNA regulates the transcriptional factor (TF), and LncRNAs means the nodes of LncRNA. Also, we count and record the number of proteins and protein–protein interactions (PPIs).

## Appendix B

BindingDB is a well accessible database of measured binding affinities, focusing chiefly on the interactions among small molecules and proteins. It provided about one million binding data for thousands of small molecules and proteins. By the following five criteria, we collected a binary classification dataset with 33,777 samples for positive examples and 27,493 for negative instances.

1. The chemical identifier (PubChem CID) is recorded, and the small molecule has chemical structure expressed by SMILES (Although both SMILES and InChI are recorded in BindingDB dataset, SMILES is easier to read and more supported by software).
2. The protein identifier (Uniprot ID) is recorded, and the protein is represented by the sequence in Fasta format.
3. The half maximal inhibitory concentration (IC50) value, a primary measure of binding effectiveness, is recorded
4. The chemical molecule weight is less than 1000 Da due to our focus on small molecule drugs.
5. According to the activity threshold discussed by Wang et al. [82], it is recorded as positive if the IC50 is less than 100 nm and negative if IC50 is greater than 10,000 nm.

Since most drug–target interaction databases do not provide real negative examples, it is a common solution to randomly sample a small number of unknown interactions as negative examples. However, unknown interactions do not mean negation, i.e., without interactions. There might just be no experimental evidence or record at present. Therefore, we followed the above procedure to evaluate and classify BindingDB samples more practically.
